# Gpr125 is a unifying hallmark of multiple mammary progenitors coupled to tumor latency

**DOI:** 10.1038/s41467-022-28937-x

**Published:** 2022-03-17

**Authors:** Elena Spina, Julia Simundza, Angela Incassati, Anupama Chandramouli, Matthias C. Kugler, Ziyan Lin, Alireza Khodadadi-Jamayran, Christine J. Watson, Pamela Cowin

**Affiliations:** 1 Department of Cell Biology, New York University School of Medicine, New York, USA; 2 Department of Dermatology, New York University School of Medicine, New York, USA; 3 Division of Pulmonary and Critical Care Medicine, New York University School of Medicine, New York, USA; 4 Department of Applied Bioinformatics, New York University School of Medicine, New York, USA; 5 Department of Pathology, University of Cambridge, Cambridge, UK

**Keywords:** Breast cancer, Cell adhesion, Mammary stem cells

## Abstract

Gpr125 is an orphan G-protein coupled receptor, with homology to cell adhesion and axonal guidance factors, that is implicated in planar polarity and control of cell movements. By lineage tracing we demonstrate that Gpr125 is a highly specific marker of bipotent mammary stem cells in the embryo and of multiple long-lived unipotent basal mammary progenitors in perinatal and postnatal glands. Nipple-proximal Gpr125+ cells express a transcriptomic profile indicative of chemo-repulsion and cell movement, whereas Gpr125+ cells concentrated at invasive ductal tips display a hybrid epithelial-mesenchymal phenotype and are equipped to bind chemokine and growth factors and secrete a promigratory matrix. Gpr125 progenitors acquire bipotency in the context of transplantation and cancer and are greatly expanded and massed at the pushing margins of short latency MMTV-Wnt1 tumors. High Gpr125 expression identifies patients with particularly poor outcome within the basal breast cancer subtype highlighting its potential utility as a factor to stratify risk.

## Abstract (web summary)

Gpr125 has emerged as a specific marker of mammary stem cells and basal progenitors. Here they show that Gpr125 cells congregate at ductal tips during morphogenesis and amass at tumor margins, and that high Gpr125 predicts early tumor onset and poor outcome in basal breast cancer.

## Introduction

Adhesion G-protein coupled receptors (GPCRs) form the second largest GPCR subfamily, yet are currently the least understood^1,2^. One member, Gpr125, resembles immunoglobulin-like cell adhesion molecules (Ig-CAM) and has leucine-rich repeats (LRR) that are found in the axonal guidance factor, Slit1, and the hair follicle progenitor marker, LRIG^1,2^. Recently, we discovered that Gpr125 identifies myoepithelial progenitors at the migrating tips of embryonic lacrimal ducts, and others have studied it as a marker of spermatogonial progenitors^3,4^.

Gpr125 is hypothesized to signal via non-canonical routes through interactions with PDZ proteins involved in cell junctions, polarity, directional movement, and morphogenesis. In zebrafish (*Danio rerio*), it clusters Disheveled into membrane subdomains and modulates planar cell polarity complexes directing convergent extension and facial motor neuron migration^5^. It is implicated in cancer through association with Discs large (Dlg), a tumor suppressor member of the ZO-1 protein family, and high Gpr125 expression has been correlated with good outcomes in colon cancer and poor outcome in myeloid leukemia^6,7^. Recombinant Gpr125 is constitutively internalized to endosomes in cultured cells suggesting a role in receptor recycling^8^. Collectively, these studies suggest that Gpr125 demarcates cells with stem/progenitor potency that participate in polarity and adhesive events linked to directed cell migration, Wnt signaling, and cancer. Here we set out to study Gpr125 in the mammary gland and mammary cancers.

Mammary glands provide an ideal system to study developmental processes in vivo. Between embryonic days 10-12 (E10-12) ectodermal cells rearrange into placodes that ~E15 commit to a mammary fate and sprout towards and invade the mammary fat pad where they branch to form a small tree^9^. Mammary development continues postnatally. The permanent ductal system is established during puberty through hormone-induced proliferation within multilayered terminal end buds (TEBs)^10–12^. Mature mammary ducts comprise a bilayered epithelium. The internal luminal layer surrounding a hollow lumen consists of hormone receptor positive and negative cells that express keratin (K) K8 and K18, and is encapsulated by a basal layer expressing smooth muscle actin (SMA), K14, and K5^13^. During pregnancy, side-branches emerge and produce alveoli at their tips that differentiate to create a functional lactating gland by birth^13,14^. Upon weaning, the gland involutes, removing the temporary, and now redundant, side-branches and alveoli while retaining the permanent ductal system and regenerating the fat pad^15^. Thus, three functional types of stem/progenitor cells support the natural life-cycle of the mammary gland: embryonic stem cells generate the mammary rudiment, pubertal progenitors produce the permanent ductal system, and long-lived adult progenitors sustain the cycles of development and destruction that are repeated with each pregnancy^16^.

Early seminal experiments demonstrated that fragments of mammary epithelium, taken from any part of the gland, can regenerate an entire mammary tree when transplanted into a fat pad cleared of its endogenous epithelium^17,18^. Serial passage of fragments or barcoded mammary cells provided evidence of a mammary hierarchy, with fully potent stem cells at the apex giving rise to more restricted ductal and alveolar progenitors^17,18^. These pioneering studies paved the way for similar analyses of the regenerative multipotency of cell subpopulations, defined by high integrin expression, termed mammary repopulating units (MRU) or mammary stem cells (MaSCs)^19,20^. Lineage tracing and single-cell RNA sequencing (scRNAseq), however, uncovered disparities between physiological stem/progenitor potency and plasticity acquired in the regenerative context^16,19–29^. There is agreement among these studies that the mammary hierarchy begins with multipotent ectodermal stem cells which during embryogenesis give rise to bipotent MaSCs that subsequently generate long-lived unipotent luminal- and basal-restricted mammary progenitors. However, the developmental timing of this potency restriction remains a source of debate^16,22–25,29–32^.

A major gap in our knowledge concerns the location of stem and progenitor populations. Attempts to address this problem produced a conundrum by identifying multiple molecularly distinct and mutually exclusive cell populations located at disparate sites^16,33–35^. For example, Lgr5+ cells are restricted to the nipple zone, whereas s-SHIP+ cells are confined to ductal tips and branches, and Procr+ and Bcl11b+ cells are dispersed along ductal borders^33–36^. Moreover, the expression of these markers in additional mammary cell types limits their usefulness as indicators of stem/progenitor cells. To date, no specific unifying progenitor hallmark has been identified. Here we show that Gpr125 identifies and locates long-lived progenitors at multiple sites and stages of mammary development. During development, Gpr125 is expressed in cells at invading ductal tips that co-express promigratory extracellular matrix (ECM) molecules as well as transcription factors that maintain a hybrid epithelial-mesenchymal cell fate and promote cellular reprogramming and invasive growth^37–41^. Our data reveal that Gpr125+ cells are expanded in murine mammary tumors arising with reduced tumor latency and that high Gpr125 expression is associated with particularly poor outcomes in basal-type breast cancer.

## Results

### Gpr125 expression is developmentally regulated

As nothing is known about Gpr125 in mammary tissue we began by analyzing mRNA levels over the course of mammary development by qRT-PCR (Supplementary Fig. 1a). Gpr125 mRNA was raised during puberty, decreased as mice reached maturity (12 weeks), was higher in earlier compared to later stages of pregnancy and peaked as the gland involuted. This temporal pattern indicates that Gpr125 mRNA is elevated when the gland is actively remodeling during ductal elongation, side-branching, and involution, and decreases as the gland differentiates.

**Fig. 1:**
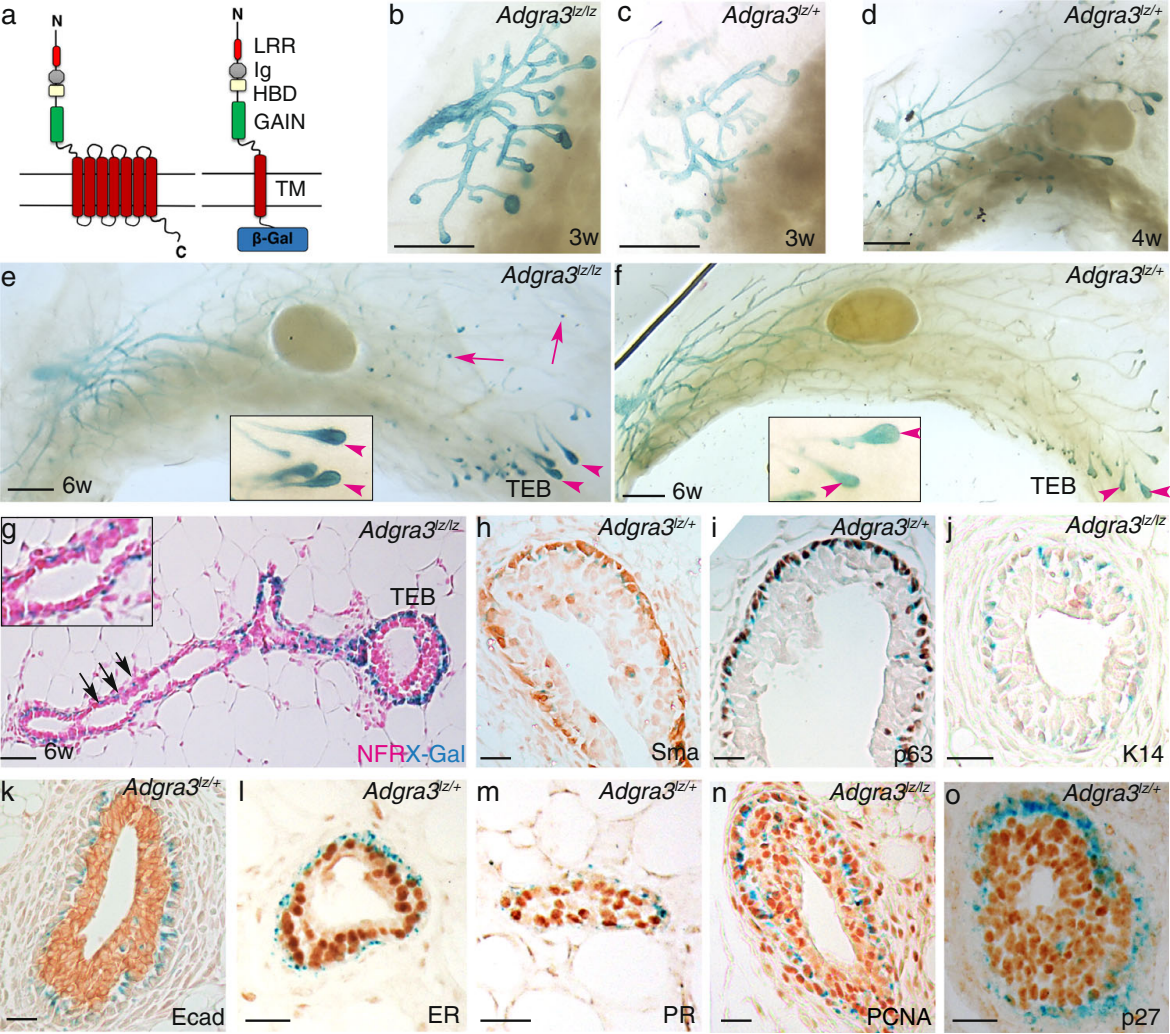
Gpr125 is expressed at predicted sites of stem/progenitor activity during pubertal mammary development. **a** Left: Schematic of Gpr125 protein, Right: Gpr125-β-gal fusion protein. N-terminus (N), leucine-rich repeats (LRR), immunoglobulin-like domain (Ig), hormone-binding domain (HBD), GPCR autoproteolytic-inducing (GAIN) domain, transmembrane region (TM), and cytoplasmic region (C). **b**–**f** Gpr125-β-gal expression in mammary whole mounts from pre-pubertal (3w *n* = 5) and pubertal (4w *n* = 4) (5w *n* = 2) and (6w *n* = 11) nulliparous mice. Scale bar = 2 mm. **g** Section of X-gal (blue) stained mammary whole-mount counterstained with nuclear fast red (NFR), shows Gpr125 expression in the cap cell layer of terminal end buds (TEB) and in cells dispersed along the basal layer of subtending ducts (arrows) *n* = 4. The inset box is a higher magnification of the area indicated by arrows. **h**–**m** X-gal (blue) stained sections of TEB with immunolocalization (brown stain) of (**h**–**j**) basal markers: smooth muscle actin (SMA), p63, Keratin (K14); Note the occasional cells expressing Gpr125 within the body layer all express basal cap cell markers; **k**–**m** luminal markers: E-cadherin (Ecad), estrogen receptor (ER), progesterone receptor (PR); **n**, **o** markers of proliferative status: (**n**) proliferating nuclear cell antigen (PCNA) and (**o**) p27. Scale bar = 50 µm. *n* = 3 mice (**h**–**k**, *n* = 3, **l**, **m**, **o**, *n* = 2 mice/antigen).

To locate Gpr125 protein expression, we X-gal stained tissue from *Adgra3*^*lz/+*^ mice in which β-galactosidase (β-gal) is fused to the first transmembrane region of Gpr125 (Fig. 1a, Supplementary Fig. 1b, d). *Adgra3*^*lz/+*^ mice are viable, fertile, and indistinguishable from wildtypes (Supplementary Fig. 1e). *Adgra3*^*lz/lz*^ mice display mild impairment in ductal elongation during early puberty (Supplementary Fig. 1f, g) but later stages of mammary development are unaffected (Supplementary Figs. 1e, h–l and 2) and dams can lactate. Both genotypes show identical patterns of reporter expression. Gpr125-β-gal was expressed throughout the dormant pre-pubescent mammary tree (Fig. 1b, c). As pubertal ductal elongation began, the X-gal staining pattern partitioned. Weak staining was retained in nipple-proximal ducts (Fig. 1d–f) and persisted there throughout postnatal development. In contrast, robust Gpr125-β-gal expression appeared in proliferative TEBs (Fig. 1d–f). This became reduced to an intense dot at ductal tips when the TEB reached the edge of the fat pad and regressed (arrows Fig. 1e). In histological sections, Gpr125-β-gal was found in cap cells (Fig. 1g) and also in single cells dispersed among the basal layer of mature ducts (Fig. 1g, inset). Gpr125-β-gal localized in cells expressing SMA, p63, and low K14 (Fig. 1h–j) and was absent from cells expressing E-cadherin (Ecad), estrogen receptor (ER), and progesterone receptor (PR) (Fig. 1k–m). Co-expression of Gpr125-β-gal with proliferating cell nuclear antigen (PCNA) and exclusion of p27 indicated their proliferative potential (Fig. 1n, o).

We interrogated the effect of hormonal deficiency and supplementation on the proliferation of Gpr125+ cells (Supplementary Fig. 3). Ovariectomized mice impaired in TEB formation, failed to upregulate expression of Gpr125 at ductal tips or to elongate the ductal system (Supplementary Fig. 3a), and showed very low levels of proliferation as detected by 5-ethynyl-2′-deoxyuridine (EdU) incorporation (Supplementary Fig. 3e). Delivery of estradiol to young ovariectomized mice restored TEB formation, ductal outgrowth, and Gpr125 expression (Supplementary Fig. 3b). EdU incorporation (Supplementary Fig. 3f–h) indicated proliferation in both Gpr125+ cap cells (gray stain arrowheads) as well as Gpr125- body cells (asterisks). Mammary glands from adult ovariectomized mice were quiescent (Supplementary Fig. 3c) but administration of estradiol and progesterone-induced pregnancy-like arborization with Gpr125 expression at branch tips (Supplementary Fig. 3d). As Gpr125+ cells do not express hormone receptors (Fig. 1k–m) we conclude that their proliferation is stimulated indirectly by hormonal action on neighboring luminal cells.

### Pubertal Gpr125+ cells display a regenerative cell profile

**Fig. 2:**
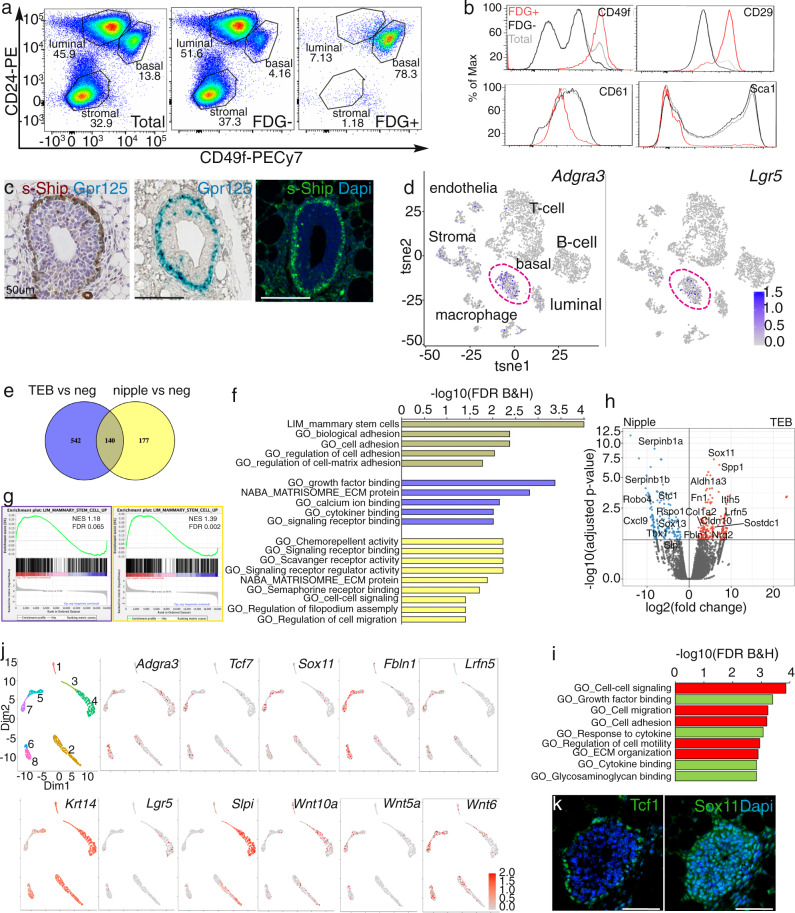
Gpr125+ cells have a MaSC/MRU profile and encompass distinct progenitor populations. **a** Representative FACS dot plots of total MECs (left) isolated from 6-week *Adgra3*^*lz/+*^ pubertal mice stained with antibodies against CD24 and CD49f. *n* = 4 mice/ experiment and repeated five times. The basal population is depleted in FDG‒ populations (center). Gpr125+/FDG+ cells gated within the CD24^med/low^/CD49f^+/hi^ basal population (right). **b** Histograms showing expression of CD49f, CD29, Sca1 and CD61 in Gpr125+/FDG+ (red line), Gpr125^‒^/FDG^‒^(black line) and total MECs (gray line) *n* = 4 mice/experiment repeated three times. **c** Detection of reporters in the cap cell layer of TEBs: left panel: co-expression of s-SHIP-EGFP detected by immunohistochemistry (brown), on top of Gpr125-β-gal detected by X-gal (dark blue) counterstained with hematoxylin (purple); and individual expression in center panel: Gpr125-β-gal detected by X-gal (blue); right panel: s-SHIP-EGFP fluorescence (green). Scale bar = 50 µm (*n* = 2 mice/panel). **d** *Adgra3* and *Lgr5* mRNA expression in the basal cell cluster, visualized by t-SNE plots extracted from the Tabula Muris dataset^42^. **e**–**g** RNA-seq based transcriptomic comparison of FACS-sorted Gpr125+ with Gpr125‒ cells isolated from TEB-distal (purple) and nipple-proximal regions (yellow) of pubertal *Adgra3*^*lz/+*^ mammary glands. **e** Venn diagram shows 140 differentially expressed genes (gray) are shared by TEB-distal and nipple-proximal Gpr125+ cells (twofold change, FDR = < 0.1). Biological significance was evaluated by (**f**) gene ontology (GO) and (**g**) gene set enrichment analysis (GSEA) analysis of TEB-distal and nipple-proximal Gpr125+ cells compared to their Gpr-negative basal cell counterparts. GSEA shows a correlation between genes highly expressed in TEB-distal (blue) or nipple-proximal (yellow) Gpr125+ cells compared to the ‘Lim_mammary_stem_cell_UP’ gene signature. **h** Volcano plot comparing gene expression of nipple-proximal Gpr125+ cells and TEB-distal Gpr125+ including highlighted candidates in nipple Gpr125+(blue dots) and TEB cells (red dots) (FDR = 0.1). **i** GO analysis yielded gene enrichment in biological processes (red bars) and molecular function (green bars) that characterize Gpr125+ cells (FDR B&H). **j** Differently expressed genes between TEB-distal Gpr125+ and nipple-proximal Gpr125+ cells were confirmed and mapped to distinct basal cell clusters by scRNAseq analysis of the pubertal dataset of Pal et al.^28^. KNetL plots are shown for cell populations expressing *Adgra3*, *Krt14*, *Tcf7, Sox11, Fbln1, Lrfn5*, *Lgr5, Slpi Wnt10a, Wnt5a, Wnt6*. **k** Distal Sox11 expression in TEBs is detected in Tcf1^+^ cap and Tcf1^‒^ body cells and some adjacent mesenchymal cells. (*n* = 4 mice). Scale bar = 50 µm. Source data are provided as a Source Data file.

To identify the subset of basal cells expressing Gpr125-β-gal we performed flow cytometry on suspensions of total mammary epithelial cells (MECs) from 6-week-old pubertal *Adgra3*^*lz/+*^ mice using a fluorogenic β-gal substrate: Fluorescein di-β-D-galactopyranoside (FDG) (Fig. 2a). Gating for Gpr125+/FDG+ cells enriched for cells within the basal (CD24^med/low^CD49f^+/Hi^) population (right panel); FDG^_^ cells were concomitantly depleted within this gate (center panel). Of note, within the basal population, Gpr125+/FDG+ cells displayed the highest level of integrins α6 and β1 (CD49f and CD29) (Fig. 2b), which are defining hallmarks of regenerative MRU/MaSCs^19,20^. Conversely, Gpr125+/FDG+ cells were low for the luminal progenitor marker CD61 (integrin β3) and negative for Sca-1, which is expressed on more committed cell types^19^. These data show that Gpr125 is expressed in cells towards the apex of the mammary hierarchy.

Next, we generated *Adgra3*^*lz/+*^;*s-SHIP-*EGFP double mutants and detected co-expression of their respective reporters by immunofluorescence in all TEB cap cells (Fig. 2c). To examine Gpr125 co-expression with Lgr5, we mined scRNAseq datasets of embryonic, pubertal, and adult MECs (Fig. 2d, Supplementary Fig. 4)^27,28,30,42^ and found a subset of cells expressing Gpr125 mRNA co-clustered with Lgr5 populations. Collectively, these results indicate that Gpr125 is expressed in disparate progenitor populations with documented regenerative capacity.

### Transcriptomic profiling of Gpr125 subpopulations

To gain further insight into the transcriptional profile of Gpr125+ cell populations, we performed whole-genome RNAseq on Gpr125+ (FDG+/CD49^hi^) and Gpr125- (FDG-/CD49^hi^) cells isolated from TEB-distal and nipple-proximal mammary regions of pubertal mice expressing the Gpr125-β-Gal. When compared to their Gpr125-negative counterparts, TEB-distal and nipple-proximal populations shared a core of 140 differentially expressed genes (Fig. 2e, Venn Diagram). Gene Ontology (GO) analysis revealed enrichment within this common core of genes encoding proteins involved in cell adhesion and regulation of cell-matrix adhesion (Fig. 2f histogram gray). The TEB-distal Gpr125+ population was enriched in the expression of genes encoding proteins involved in growth factor, calcium, and cytokine binding and ECM (Fig. 2f histogram purple), whereas the nipple-proximal Gpr125+ cells were enriched in the expression of cell-cell signaling, cell migration and guidance and chemo-repulsion factors (Fig. 2f histogram yellow). Gene set enrichment analysis (GSEA) confirmed that both TEB-distal and nipple-proximal Gpr125+ populations are significantly enriched in the Mammary Stem Cell-UP gene signature derived from MRU/MaSC cells (Fig. 2g). When we compared TEB-distal and nipple-proximal Gpr125+ cells to each other (Fig. 2h, volcano plot and 2i) TEB-distal Gpr125+ cells showed significantly increased expression of mRNA encoding stem cell markers such as Aldh1a3, promigratory cell-cell and cell-matrix factors such as fibronectin, fibulin1, as well as the homophilic cell adhesion molecule, Lrfn5 that promotes neurite outgrowth. Of note, the highest expressed gene in this population was *Sox11* (SRY-related high-mobility-group (HMG) box 11), which encodes a transcription factor implicated in breast cancer invasion, that is responsible for keeping cells in a hybrid epithelial-mesenchymal state and is a key regulator of stem and progenitor cells^38–41^. In contrast, nipple-proximal Gpr125+ cells showed high expression of genes encoding protease inhibitors Serpin and Slpi that maintain elastin integrity as well as chemotactic guidance factors, such as Robo4, and Cxcl9 and the Wnt pathway agonist, Rspo1.

To confirm specific differences in gene expression between the two pubertal Gpr125 subpopulations, we analyzed the pubertal scRNAseq dataset of Pal et al^28^. While t-SNE plots partitioned the basal population into two groups (Supplementary Fig. 4b), KNetL dimensionality reduction^43^ was able to resolve eight clusters permitting more detailed observation (Fig. 2j and Supplementary Fig. 4c). *Acta2* encoding the basal marker SMA, was expressed in all 8 clusters (Supplementary Fig. 4c). *Adgra3* was induced in clusters 4–7 (Fig. 2j). *Lgr5* expression, which is restricted to nipple-proximal cells, was found in cluster 4 whereas *Sox11* and *Tcf7* were expressed in clusters 5–8 (Fig. 2j). To confirm distal expression Sox11 and Tcf1 (encoded by *Tcf7)* at the protein level we performed immunofluorescence microscopy. Tcf1 was found exclusively within the nuclei of cap cells. Sox11 showed prominent nuclear localization in both cap and body cells of the TEB as well as in some neighboring stromal cells (Fig. 2k). In keeping with the role of Sox11 in maintaining a hybrid epithelial-mesenchymal cell state, we noted that clusters 5–8 showed significantly lower levels of *Krt14* and *Krt5* expression than clusters 1–4 (Fig. 2j, Supplementary Fig. 4c). The KNetL plots confirmed *Slpi* expression specifically in nipple-proximal cluster 4, expression of *Lrfn5* and *Fbln1* specifically within TEB-distal clusters and also found distinct expression patterns of genes encoding Wnt ligands: *Wnt10a* was expressed in nipple-proximal cluster 4 and *Wnt5a* and *Wnt6* in the TEB-distal clusters 5–8 (Fig. 2j). Collectively, our analyses show that Gpr125 encompasses distinct progenitor populations in the mammary gland that share a common gene expression core as well as site-specific transcriptional profiles.

### Pubertal Gpr125+ cells are unipotent basal progenitors

**Fig. 3:**
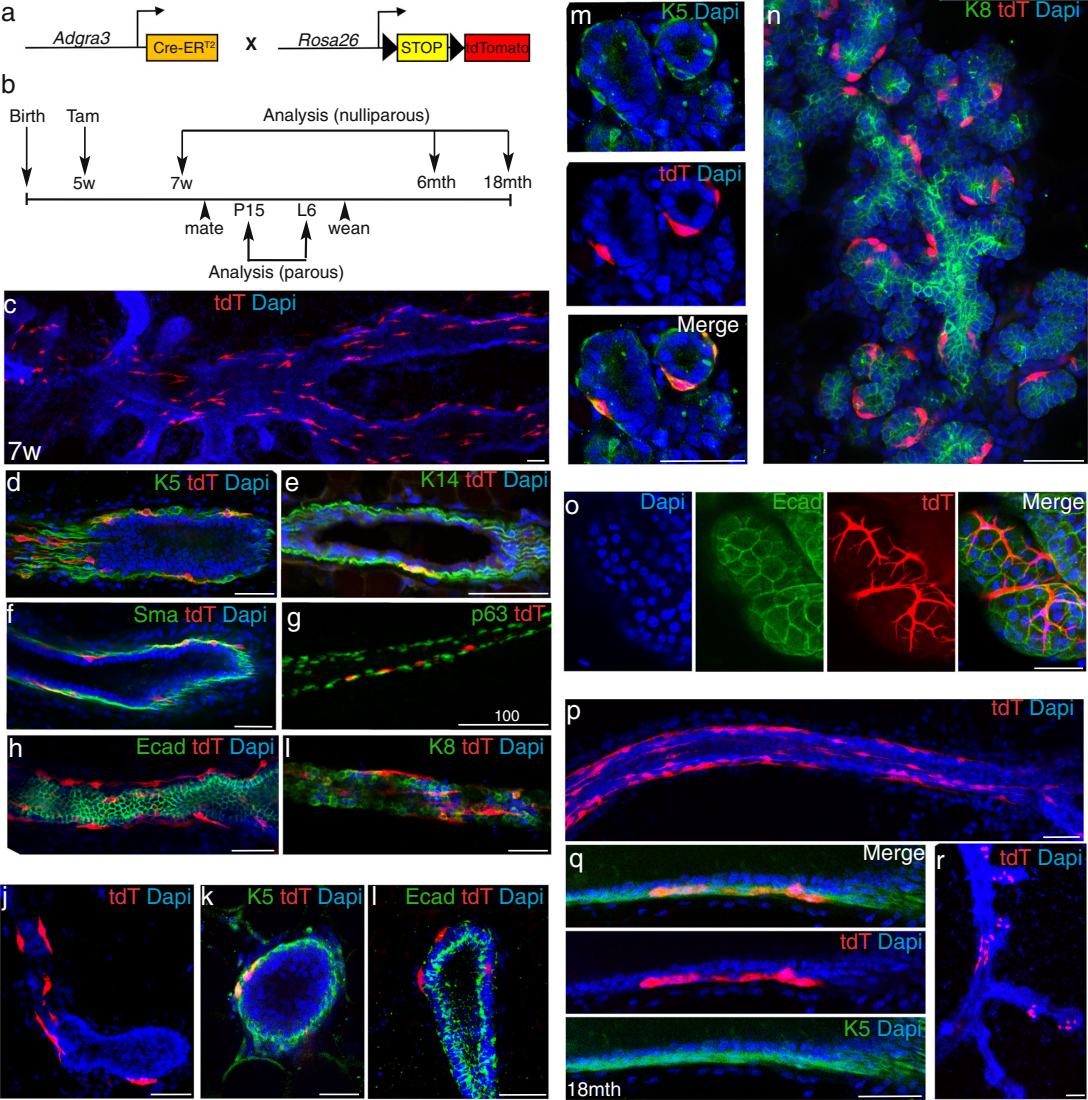
Pubertal Gpr125+ cells are long-lived unipotent basal progenitors. **a** Genetic strategy used to target *tdTomato* expression to *Gpr125/Adgra3*-expressing cells and their progeny. **b** Schematic illustrating strategy to trace the lineage of cells that express Gpr125 during early puberty by delivering Tamoxifen (Tam) to (5w) nulliparous mice and harvesting glands during late puberty (7w), pregnancy (P15); lactation (L6), and in aged and multiparous mice. **c** Representative 3D-imaging of mammary gland nipple-proximal region of *Adgra3-CreER*^*T2*^*;tdT* mice analyzed 2 weeks after labeling. **d**–**g** tdT+ cells with immunolocalization (green) of basal markers K5, K14, SMA, and p63, and exclusion of luminal markers Ecad, K8 (**h**, **i**). **j** tdT+ cells in the cap cell layer of TEB and subtending duct with immunolocalization of basal K5 (**k**) and exclusion of luminal Ecad (**l**). **m** p15 Immature alveoli show basally located tdT+ cells that express K5, but lack K8 expression (**n**). **o** tdT+ cells form characteristic basket-like morphology of mature myoepithelial cells enmeshing an Ecad+ alveolus in the lactating mammary gland. **p**–**r** Extensive strips of tdT+ cells remain along the outer basal layer of ducts in aged mice (**p**, **q**) and after multiple pregnancies (**r**). Scale bar = 50 µm. tdT = red, tdT+ cells; DAPI = blue nuclear staining. Two glands from each of the five mice were analyzed/stage.

To position pubertal Gpr125+ cells within the mammary hierarchy, we carried out lineage tracing to determine their physiological potency. We generated a mouse strain harboring a *CreER*^*T2*^ module inserted after the endogenous *Adgra3* promoter (Fig. 3a and Supplementary Fig. 1b, c), crossed them to the Tomato (tdT) lineage reporter strain (*B6.Cg-Gt(ROSA)26Sor*^*tm14(CAG-tdTomato)Hze/J*^) and initiated tracing in 5-week-old female progeny by delivering Tamoxifen (Tam) via intraperitoneal (IP) injection (Fig. 3b). After two weeks we observed clusters of tdT+ cells within the basal layer of ducts (Fig. 3) that were most abundant in the nipple-proximal region (Fig. 3c). tdT+ cells displayed the characteristic bipolar shape of myoepithelial cells and by immunofluorescence, co-localized with the basal markers K5, K14, SMA, and p63 (Fig. 3d–g) and were devoid of the luminal markers Ecad and K8 (Fig. 3h, i). tdT was also found in cap cells of TEB (Fig. 3j–l) and in strips of cells extending down the basal surface of subtending ducts.

Next, we mated mice in which tracing had been initiated during puberty and analyzed their glands during pregnancy. Again, tdT+ cells were basally restricted. At p15.5 clusters of tdT+ cells colocalized with K5-expressing cells (Fig. 3m) surrounding immature Ecad+ alveoli (Fig. 3n). At lactation day 6 (L6), tdT+ cells displayed the typical basket-like features of contractile myoepithelial cells, enmeshing fully differentiated Ecad+ alveoli (Fig. 3o, Supplementary movie 1).

### Gpr125+ cells are long-lived progenitors

We addressed the longevity of the Gpr125+ progenitors labeled during puberty by tracing their tdT+ progeny in both aged nulliparous mice (Fig. 3p, q) and multiparous mice (Fig. 3r). In both, clusters and extensive strips of elongated tdT+ cells were found along basal ductal borders. Collectively, our lineage tracing results reveal that Gpr125 identifies unipotent basal progenitors present in the permanent ductal system during puberty that are long-lived and retain progenitor activity after multiple pregnancies and throughout the average lifespan of the mouse.

### Gpr125+ cells congregate at leading tips of side branches

**Fig. 4:**
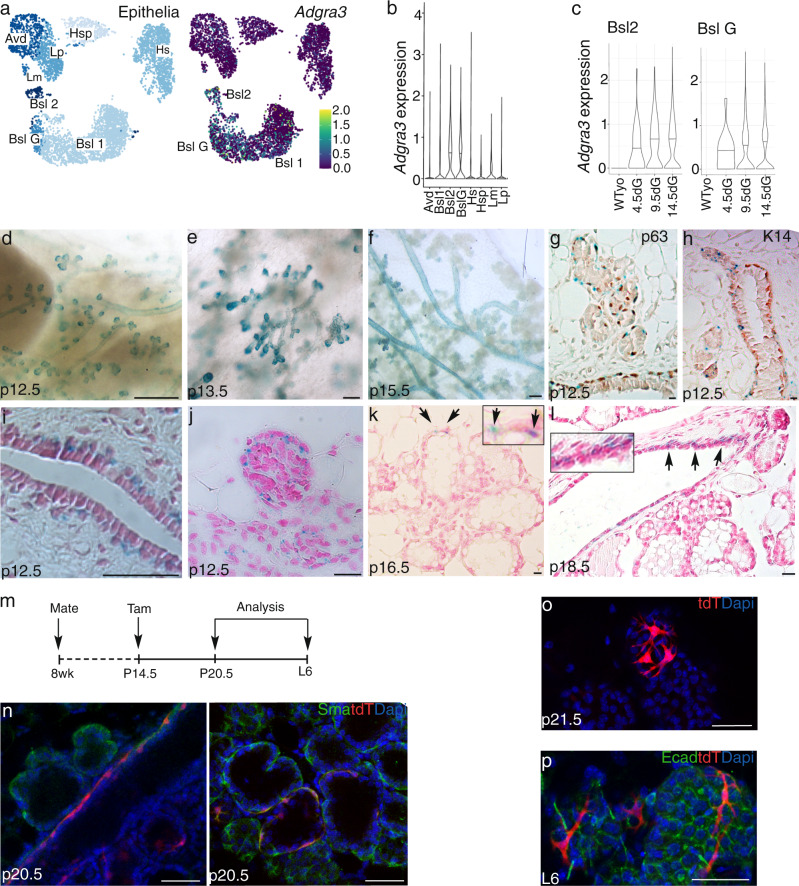
Cells expressing Gpr125 on leading tips of branches during pregnancy are unipotent basal progenitors. **a** UMAP plot and violin plot (**b**) showing *Adgra3* expression restricted to the mammary basal populations. **c** Violin plot showing expression levels at days of gestation (dG) as indicated in Basal 2 and Basal G cell types^44^. **d**–**f** X-gal (blue) stained mammary whole mounts from pregnant mice Scale bar = 500 µm (*n* = 4 mice/stage) showing Gpr125-β-gal expression at **d** sites of emerging side-branches at 12.5 days of pregnancy (p12.5), **e** tips of elongating side-branches at p13.5, and **f** in ducts but not alveoli at p15.5. **g**, **h** X-gal (blue) stained sections of a permanent duct and side branch with immunolocalization (brown) of basal marker p63 and K14. Scale bar = 20 µm (*n* = 2 mice). **i**–**l** X-gal (blue) stained sections counterstained with NFR. Boxed insets are higher magnification of regions indicated by arrows (*n* = 2 mice/stage). **m** Schematic illustrating strategy to trace the lineage of cells that express Gpr125 during early pregnancy by delivering Tam at p14.5 and harvesting glands at p20.5 and L6 mice. **n** tdT+ SMA+(red/green) cells were found in ducts and alveoli at p20.5 and at birth (**o**). **p** Fully differentiated tdT+ cells were devoid of luminal Ecad marker (green) at L6. Scale bar = 50 µm. tdT = red, tdT+ cells; DAPI = blue, nuclear staining. One gland from each of the five mice was analyzed.

To assess *Adgra3* mRNA during pregnancy we examined the scRNAseq data of Bach et al. focused on gestational changes in wildtype cells and found *Adgra3* mRNA is highly upregulated in Bsl2 and BslG populations beginning ~p4.5 and peaking ~p14 (Fig. 4a–c)^44^. *Adgra3* appears at #23 in the list of differentially expressed marker genes for Bsl2 and #63 for BslG population (https://crukci.shinyapps.io/brca1tumourigenesis/)^44^.

To investigate Gpr125 protein expression during early pregnancy (p12-13.5) we again detected Gpr125-β-gal expression by X-gal staining. Gpr125-β-gal appeared focally where side-branches emerge from ducts and concentrated at branch tips (Fig. 4d–e, g–j). X-gal staining was prominent along the basal layer of permanent ducts during late pregnancy but was absent from differentiating alveoli (Fig. 4f, k, l) with the exception of rare Gpr125-β-gal+ cells that likely represent the branch tip of each alveolar cluster (Fig. 4k arrowheads).

To interrogate the potency of Gpr125+ cells present during pregnancy we initiated lineage tracing in mid-pregnant mice at p13.5 and analyzed their glands just prior to birth and during lactation (Fig. 4m). Again, tdT+ cells exclusively displayed myoepithelial characteristics (Fig. 4n–p).

Having established that Gpr125 cells are unipotent during postnatal mammary development, we next assessed their plasticity in a regenerative setting by using a lineage tracing approach combined with a transplantation assay. We induced lineage tracing by delivering Tam to 5-week-old mice then two weeks later harvested and prepared MECs from one of their #4 inguinal glands and transplanted them into *FoxN1*^*nu*^ recipients (Supplementary Fig. 5a). Glands were harvested from both the donor and host after two and three weeks and their labeled cell progeny were compared. As expected the Gpr125 lineage in glands remaining in the donor was exclusively basal (Supplementary Fig. 5b–d). In contrast, a mixed basal and luminal lineage was generated within the reconstituted trees of transplant recipients (Supplementary Fig. 5e) indicating that in the context of experimentally induced regeneration Gpr125+ cells regain bipotency.

### Gpr125 detects embryonic bipotent and unipotent progenitors

**Fig. 5:**
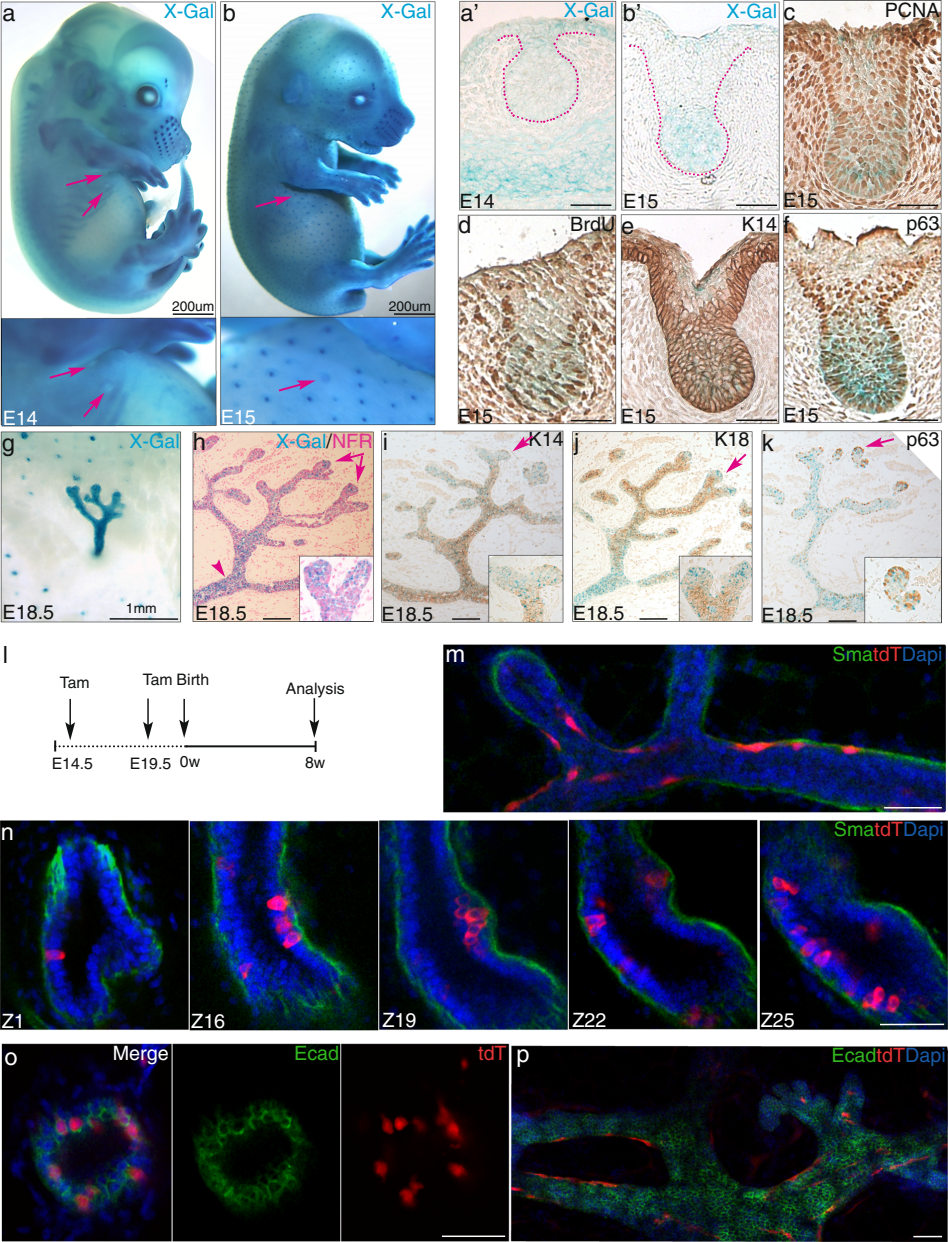
Gpr125 identifies an early bipotent and later unipotent basal progenitor population during embryogenesis. **a**, **b** X-gal (blue) stained *Adgra3*^*lz/+*^ embryos at embryonic day (E)14 (**a**) and E15 (**b**). Arrows indicate mammary buds and sprout respectively magnified in boxes below. Scale bar = 200 µm (*n* = 2 litters/stage). **a**’–**f** Gpr125-β-gal expression in sections of E14 buds (**a**’) and E15 sprouts (**b**’–**f**), with immunolocalization (brown) for proliferative markers PCNA and BrdU (**c**, **d**), and for K14 and p63 (**e**, **f**). Scale bar = 50 µm (*n* = 2 embryos/stage). **g** X-gal (blue) stained skin whole mount showing Gpr125-β-gal expression in the E18.5 tree and hair follicles encircling the developing nipple zone. Scale bar = 1 mm. (*n* = 2 litters of embryos). **h**–**k** Sections of the E18.5 rudiment tree stained with X-gal (blue) followed by NFR counterstain (**h**) or immunochemical detection (brown) of K14, K18, and p63 (**i**–**k**). Boxed insets are higher magnification of branch tips regions indicated by arrows. Scale bar = 50 µm (*n* = 2 embryos/stage). **l** Tracing of Gpr125+ cells in E14.5 or E19.5 embryos were initiated by delivering Tam to pregnant *Adgra3*^*cre/cre*^ dams mated to *Rosa26*^*tdT*^ mice. Mammary tissue from the progeny was analyzed at 8 weeks of age. **m**–**o** 3-D images showing representative regions of pubertal ducts from E14.5 labeled embryo containing clusters of basally located tdT+ cells that co-express the basal marker SMA (green) (**m**) as well as tdT+ columnar luminal cells lacking SMA (**n**) and expressing luminal marker Ecad (green) (**o**). **p** Glands from progeny labeled at E19.5 show basally restricted tdT+ cells devoid of Ecad. tdT = red, tdT+ cells; DAPI (blue) nuclear staining. Scale bar = 50 µm. Two glands from each of the three mice were analyzed/stage.

Extending our studies to embryonic mammary development, we found that early *Adgra3*^*lz/+*^ embryos showed diffuse ectodermal Gpr125-β-gal expression that by E14.5 concentrated into ectodermal appendages, such as whisker follicles but was absent from the mammary line, placodes and buds (arrows Fig. 5a, a’). However, at E15 Gpr125-β-gal appeared in the mammary sprout (arrows Fig. 5b, b’), coincident with the onset of proliferation, indicated by nuclear PCNA and bromodeoxyuridine (BrdU) (Fig. 5c, d), and was concentrated towards the leading tip together with K14 and p63 (Fig. 5e, f). At this stage Gpr125-β-gal also became concentrated in the “bulge” stem cell compartment of hair follicles and whiskers (Supplementary Fig. 6). At E18.5 Gpr125-β-gal was strongly expressed in the rudimentary tree (Fig. 5g) and concentrated in the lactiferous duct and the multilayered branch tips (Fig. 5h). Although K14 expression was present in most cells (Fig. 5i) and K18 was increased in central cells (Fig. 5j), Gpr125+ branch tips lacked both keratins (Fig. 5i, j arrow and insets) but expressed p63 (Fig. 5k arrow and insets).

To test the potency of embryonic Gpr125+ cells we crossed *Adgra3*^*cre/cre*^ to *Rosa26*^*tdT*^ mice and administered Tam to the pregnant dams to label embryos (Fig. 5l) then analyzed these pups at 8 weeks of age. Glands from mice labeled at E14.5 revealed tdT not only in basally located bipolar cells co-expressing SMA (Fig. 5m), but also in columnar cells situated above the SMA + basal layer (Fig. 5n) that expressed the luminal marker Ecad (Fig. 5o). By contrast, when tracing was initiated at E19, tdT+ cells were exclusively basal, bipolar, and SMA+ (Fig. 5p).

These data show that Gpr125 appears at the onset of directed growth in a bipotent progenitor population expressing markers of both lineages (K14/K18). However, before birth Gpr125 cells become lineage-restricted and from thereon function as unipotent basal progenitors. Of note, Gpr125 expression concentrates at this stage in undifferentiated p63^+^K14-/K18^−^ cells at branch tips poised for a ductal extension.

### Immature Gpr125+ cells are enriched in MMTV-Wnt1 tumors

**Fig. 6:**
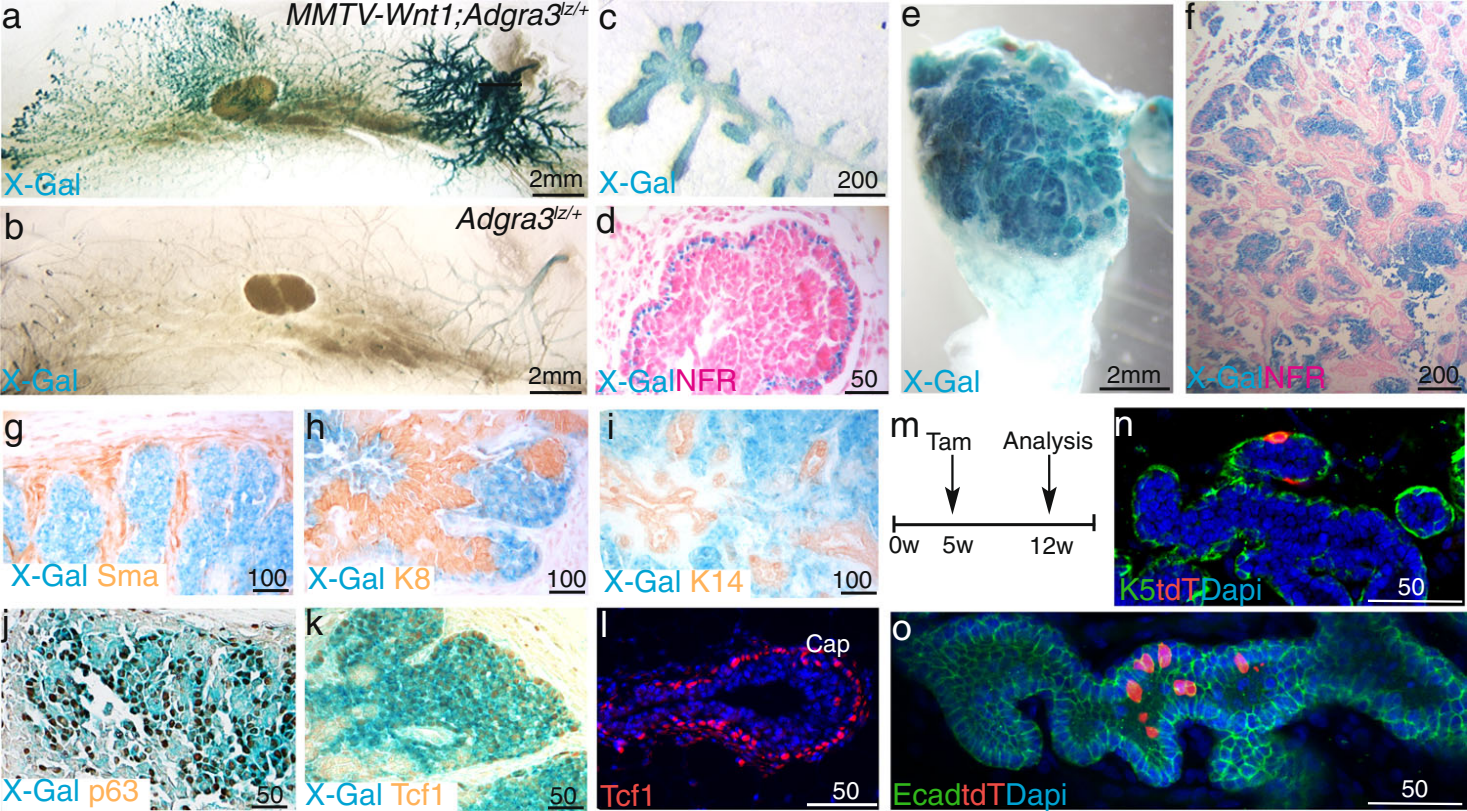
Gpr125 + progenitors are expanded in MMTV-Wnt1 tumors and retain embryonic features and bipotency. **a**–**c** X-gal (blue) stained mammary whole mounts from 8-week-old *MMTV-Wnt1;Adgra3*^*lz/+*^ mice (*n* = 23 mice) show robust Gpr125-β-gal expression in hyperbranched ductal tips (**c**) and nipple-proximal zones compared to (**b**) control *Adgra3*^*lz/+*^ littermate. **d** X-gal (blue) section of hyperbranched ductal tip counterstained with NFR presenting basal restriction of Gpr125-β-gal expression in 12-week-old *MMTV-Wnt1;Adgra3*^*lz/+*^ mice. **e**-**h** X-gal (blue) stained whole mount and sections counterstained with NFR of *MMTV-Wnt1;Adgra3*^*lz/+*^ tumor (**g**–**k**) showing Gpr125 cells devoid of immunolocalization (brown) for SMA, K14 or K8, but expressing p63 and Tcf1 (*n* = 4 tumors/marker). **l** Tcf1 expression (red) in the cap cells of normal TEB (*n* = 3 mice). **m**–**o** Lineage tracing strategy in 5-week-old *MMTV-Wnt1;Adgra3*^*lz/+*^ produced both (**n**) K5 + (green) and (**o**) Ecad + ;tdT + (green/red) cells in hyperplastic glands at 12 weeks. DAPI = blue nuclear staining. One gland from each of the five mice were analyzed.

Next, we investigated Gpr125 in mouse breast cancer models. Gpr125 showed the highest mRNA expression in MMTV-Wnt1 mice (Supplementary Fig. 7a) which develop mixed-lineage tumors enriched in cells with MaSCs profiles and show transcriptomic resemblance to basal-type breast cancer^45–49^. We, therefore, generated *MMTV-Wnt1*; *Adgra3*^*lz/+*^ mice and analyzed glands over the course of tumor progression. In 8-week-old mice, Gpr125-β-gal was expressed exclusively in basally located cells within the nipple proximal hyperplasia and on hyperbranched tips (Fig. 6a–d). Robust expression was seen in tumors, where Gpr125+ cells formed large homogenous regions and concentrated at pushing margins (Fig. 6e–h). Although Wnt-1 tumors display regions composed of K14+ and K18+ bilayers these populations rarely overlapped with Gpr125. Gpr125+ cells also lacked both SMA and K8 and although some expressed K14, the majority lacked both keratins (Fig. 6g–i). They did, however, express p63 as well as Tcf1 (Fig. 6j, k). To determine the effect of Wnt expression on the potency of Gpr125 expressing cells, we generated *MMTV-Wnt1;Adgra3*^*cre/+*^*; Rosa26*^*tdT*^ mice and performed lineage tracing by delivering Tam to 5-week old pubertal mice and harvesting hyperplastic glands from 12-week old mice (Fig. 6m). tdT was found in both SMA+ and Ecad+ cells (Fig. 6n, o). Gpr125 cell progeny were concentrated at tips of invasive branches (Supplementary movie 2). These data indicate that in the context of MMTV-Wnt1 transformation pubertal Gpr125+ cells retain the undifferentiated mesenchymal characteristics of TEB cap cells and the bipotency of embryonic progenitors.

### Gpr125 linkage to poor outcome in basal breast cancer

**Fig. 7:**
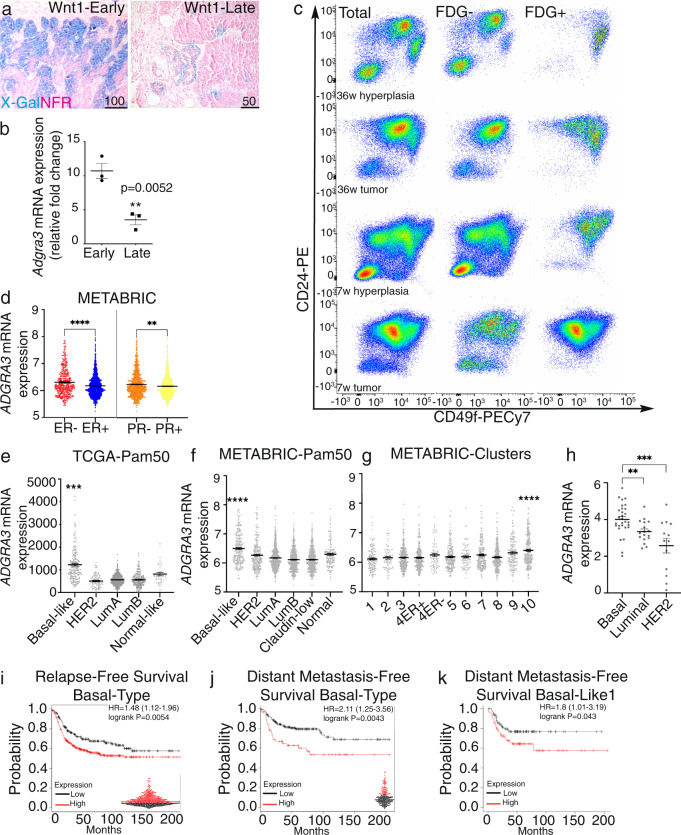
High Gpr125 expression is predictive of poor outcome. **a** X-gal/NFR stained sections showing higher Gpr125-β-gal expression in a short (7w) latency *MMTV-Wnt1;Adgra3*^*lz/+*^ tumor (Wnt1-early) versus a long (36w) latency (Wnt1-late) tumor. Scale bar 100 µm and 50 µm. (*n* = 5 tumors/subtype). **b** qRT-PCR analysis of relative *Adgra3* mRNA expression in early and long latency tumors *MMTV-Wnt1;Adgra3*^*lz/+*^ tumors (*n* = 3 tumors/subtype, **p = 0.0052). **c** Representative FACS dot plots of total MECs isolated from *MMTV-Wnt1;Adgra3*^*lz/+*^ hyperplastic uninvolved glands and associated short and long latency tumors stained with CD24 and CD49f. FDG+ cells in: 36w tumor = 13331; 36w hyperplasia = 4048; 7w tumor = 64889; 7w hyperplasia = 13156. Plots are representative of three independent experiments. **d** *ADGRA3* expression in (left) ER- (*n* = 445) and ER+ (*n* = 1459) and in (right) PR- (n-895) and PR+ (*n* = 1009) BC patient samples from the METABRIC dataset **p = 0.0042, ****p < 0.0001. **e**, **f** *ADGRA3* expression in BC patient samples from TGCA PanCancer Atlas (*n* = 1084) (**e**) and METABRIC (*n* = 2509) (**f**) datasets classified using the PAM50 geneset; ****p < 0.0001. **g** Integrative clusters within the METABRIC dataset showing high *ADGRA3* expression levels in cluster 10; ****p < 0.0001, Mann–Whitney test two tailed. **h** *ADGRA3* expression in human breast cancer cell line from Depmap database (https://depmap.org/portal/) **p = 0.0068, ***p = 0.0002, Unpaired *t* test two-tailed. **i**–**k** Kaplan–Meyer curves depicting ‘Relapse-free survival’ and ‘Distant Metastasis-Free Survival’ in months for high (red lines) and low (black lines) *ADGRA3* mRNA expressing human basal-type (**h**, **i**) and basal-like-1 TNBC (**j**) breast cancer subtypes. Auto best fit cutoff was used to divide patients into high and low expression (indicated in beehive plots) sourced from (https://kmplot.com/analysis/index.php?p=service&cancer=breast) KMplotter^75^. logrank p-value and Hazard Ratio (HR) indicate a significant association between high expression and poor prognosis. Each scatter dot plot represents the mean ± SEM. Source data for this figure are provided as a Source Data file.

Wnt1 tumors have been divided into two subtypes with distinct gene expression (ex) profiles: Wnt1-early(ex) and Wnt1-late(ex), which correlate with early (average 6.5 weeks) and late (average 22.5 weeks) tumor onset respectively^45,46^. We noted that Gpr125-β-gal expression was consistently more extensive in *MMTV-Wnt1*; *Adgra3*^*lz/+*^ tumors with shorter latency (Fig. 7a). By qRT-PCR we confirmed 3-fold higher levels of Gpr125 mRNA in *MMTV-Wnt1*; *Adgra3*^*lz/+*^ tumors with very short latency compared to those with long latency (*p* = 0.0052) (Fig. 7b). Prospective analysis of a microarray dataset for murine mammary cancer models showed that Gpr125 was highest in Wnt-Early(ex) tumors (Supplementary Fig. 7a). To investigate the course of Gpr125+ cell expansion we carried out flow cytometry. In hyperplastic glands, the Gpr125+/FDG+ population localized within the traditional basal gate but in tumors, it was expressed in a population with intermediate CD49 levels (Fig. 7c) that was more pronounced in uninvolved glands and tumors that arose early. Collectively, these data show that expansion of the Gpr125 tumor population correlates with early tumor onset in mice. We further investigated *GPR125/ADGRA3* expression in human breast cancer patients within the METABRIC and the TCGA datasets downloaded from the cBioPortal platform. *ADGRA3* mRNA level was significantly higher (****p < 0.0001, **p = 0.0042) in tumors lacking expression of hormone receptors (Fig. 7d). No correlation was found between *ADGRA3* expression and HER2 status (Supplementary Fig. 7c). *ADGRA3* expression was significantly higher (*p = 0.0322) with BRCA1 status within the METABRIC but not the TCGA datasets (Supplementary Fig. 7d, e). Elevated *ADGRA3* mRNA levels were found in higher grade tumors (Supplementary Fig. 7f). The highest expression of *ADGRA3* was found within basal-type breast cancers within in the PAM50 classification of both databases (Fig. 7e, f) and in microarray analyses (Supplementary Fig. 7b). *ADGRA3* was significantly higher in the METABRIC integrative cluster 10 (Fig. 7g), defined as a basal-like cancer enriched subgroup with high genomic instability and alteration of key cell-cycle-related genes. Expression of *ADGRA3* mRNA was higher in human breast cancer cell lines assigned to Basal A/B categories within the DepMap database (https://depmap.org/portal/) compared to those categorized as luminal or HER2-positive (Fig. 7h and Supplementary Fig. 7h). Interrogating publicly available datasets using the kmplotter algorithm showed that higher *GPR125* levels within the basal subtype correlated with worse patient outcome in terms of relapse-free survival (*p* = 0.0054) and distant metastasis-free survival (*p* = 0.0043) (Fig. 7i, j red line), and this was confirmed using the BreastMark algorithm for disease-free survival (Supplementary Fig. 7g, blue line). Last we found that particularly high *GPR125* expression correlated with poor distant metastasis free survival in the Basal-Like 1 (BL-1) triple-negative breast cancer (TNBC) subgroup (Fig. 7k).

## Discussion

**Fig. 8:**
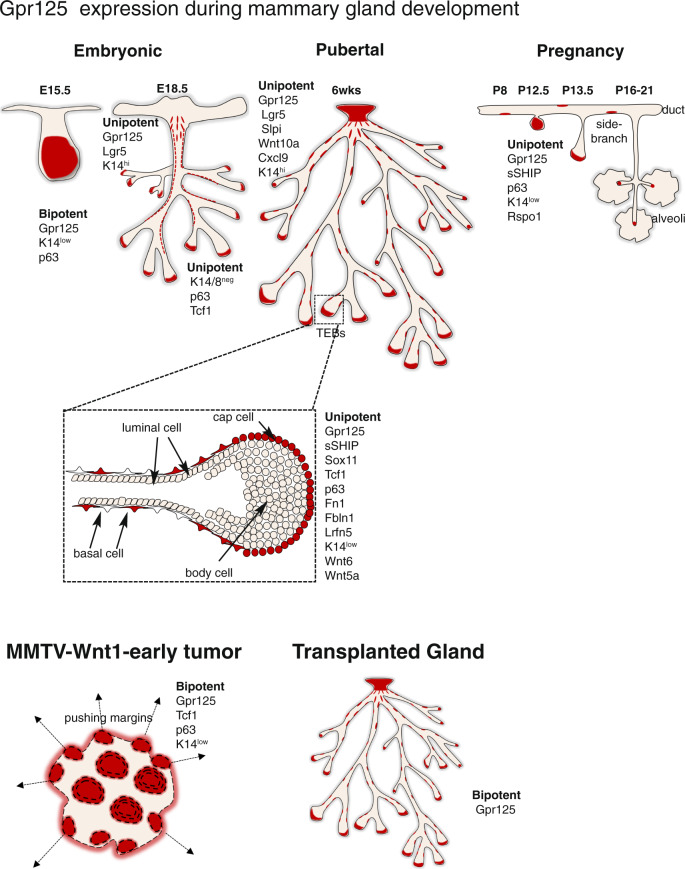
Schematic of Gpr125 progenitor location, profile, and potency characteristics over the course of mammary development. Gpr125 is expressed ~E15 in a bipotent progenitor population concentrated towards the growing tip of the mammary sprout. Later, ~E18, as lineage segregation ensues, it becomes restricted to basal unipotent progenitors confined to K14+;Lgr5+ cells in the nipple region and distal K14-/K18-/p63+/Tcf1+ cells at ductal tips. Gpr125 is retained in the nipple proximal zone throughout postnatal mammary development. During puberty is strongly expressed in cap cells of terminal end buds during ductal elongation and a population dispersed along the maturing ducts. During pregnancy Gpr125 expression increases in unipotent basal progenitors located at tips of emerging side-branches but is absent from differentiated alveoli. In MMTV-Wnt1 early tumors bipotent Gpr125 + K14-;K18- progenitors are expanded and located in large islands and at pushing margins. In transplanted glands Gpr125 progenitors regain bipotency.

Here, we have investigated Gpr125 in the developing mammary gland and breast cancer. Our results demonstrate the powerful ability of Gpr125 to localize progenitors with great specificity at multiple sites and stages of mammary development (Fig. 8). We show that Gpr125+ cells are concentrated at the invading tips of migrating ducts and branches during mammary developmental remodeling and massed at pushing margins in tumors. Our results demonstrate that Gpr125+ cells are expanded in early onset tumors in mice and similarly, that elevated Gpr125 levels in humans with basal breast cancer are predictive of particularly poor outcome in terms of earlier dissemination and relapse.

Our analyses show that Gpr125 is expressed in stem cell compartments of multiple ectodermal appendages (Supplementary Fig. 6). Together with its expression in spermatogonial progenitors this demonstrates that Gpr125 has value in demarcating early progenitors in multiple tissues^4^. By lineage tracing, we show that Gpr125 identifies a bipotent cell population in the embryonic E15 mammary bud. As scRNAseq studies found no lineage bias among mammary cells at this stage, this strongly suggests that Gpr125 recognizes MaSCs^29,30,50^. However, by E19, Gpr125-expressing cells have clearly become committed unipotent basal progenitors, a finding that is consistent with recent studies by Fre et al., indicating that lineage restriction begins early and is completed before birth^25,31^.

By lineage tracing, we demonstrate that postnatal Gpr125 cells give rise exclusively to the basal lineage. Analyses of Gpr125-β-gal expression pattern and their progeny indicate that Gpr125 identifies distinct long-lived unipotent basal progenitors at multiple locations during postnatal mammary development. Cells expressing *Adgra3* and *Lgr5* mRNAs co-cluster, and Gpr125 protein localization mirrors that of Lgr5 in the nipple-proximal zone^34^. Cap cells in the TEBs co-express Gpr125 and s-SHIP protein^33,51^ and Gpr125 expression in progenitors dispersed throughout the mature ductal system is consistent with descriptions of cells with regenerative capacity in early mammary transplantation studies and similar to that of Procr and Bcl11b^17,18,35,36,52^. Thus, Gpr125 is universal marker of disparate unipotent basal progenitor subpopulations. Our results reveal that nipple-proximal and TEB-distal Gpr125+ populations are both enriched in gene signatures described for regenerative MaSCs and share a common transcriptional core of cell-cell and cell-matrix adhesion molecules and regulators. Gpr125+ cells express the highest level of integrins of all basal cells, suggesting a strong dependency on cell-matrix attachment for progenitor phenotype and function. Several adhesion-GPCRs, for example, Gpr56/Adgrg1, which functions in cell guidance/adhesion, have been shown to interact directly with cell matrix proteins and regulate basement membrane organization^53^. Gpr125, is structurally related to Ig-CAMs, and localizes to basolateral borders between cap cells and underlying body cells. However, as is the case for most adhesion GPCRs, its potential ligands, role in cell-cell adhesion, and downstream signaling pathway remain obscure.

Our bulk RNA-seq data indicate that Gpr125+ cells at the nipple-proximal end of the gland are engaged in chemo-repulsion and the formation of cell surface structures associated with cell movement such as lamellipodia. In contrast, Gpr125+ cells at the TEB-distal tips exhibit a hybrid epithelial-mesenchymal phenotype and are equipped to bind chemokine and growth factors and secrete a promigratory matrix. It is striking that Gpr125 is concentrated at sites of directed migration in several tissues that undergo branching morphogenesis^3^. In mammary gland, these sites include the tips of the mammary sprout, rudimentary tree, pubertal TEB, and side-branches. This expression pattern together with the impairment in ductal extension observed in its absence support an involvement of Gpr125 in progenitor migration. This potential role for Gpr125 is consistent with previous reports showing Gpr125 levels influence facial motor neuronal precursor migration in zebrafish^5^. Indeed, regulation of directed cell movement may be a common function of the Adgra subfamily as the closely related protein, Gpr124, is required for tip cell function in endothelia during angiogenesis^54^.

Gpr125+ cells at the distal tips of pubertal ducts express particularly high levels of genes, such as *Sox 11, Aldh1a3,* and *Lrfn5*, associated with stemness, neurite outgrowth, and cancer, respectively. The most prominent of these is *Sox11*, which first appears during stages of intense inductive epithelial-mesenchymal signaling in the embryonic mammary bud^55,56^ and is present in embryonic mammary progenitor cell signatures^50,57^. Sox11 has been shown to reactivate an embryonic gene signature during epidermal wound healing that regulates expression of ECM and the cytoskeleton, to enhance proliferation, migration^58^. In keeping with recent studies demonstrating that Sox11 maintains a hybrid epithelial-mesenchymal cell state, our scRNAseq results show that Gpr125+ cells in TEB express very low levels of basal keratins, K5 and K14, and show strong expression of genes encoding promigratory ECM proteins, fibronectin, and fibulin1.

It is possible that the Gpr125 and Sox11 co-expression in mammary progenitors relates to their involvement in Wnt signaling, which is critical at all stages of mammary development and has been shown to sustain progenitor potency in vitro^12,26,47,48,59,60^. Gpr124 selectively promotes canonical signaling of specific Wnt ligands by stabilizing their receptor interactions at the cell surface^54^. Studies showing Gpr125 becomes constitutively internalized into endosomes when expressed in cultured cells suggest it too could function in receptor recycling^8^. In zebrafish, Gpr125 has been implicated non-canonical Wnt/planar polarity signaling^5,6^. Our scRNAseq analysis indicates *Adgra3* mRNA is co-expressed with *Wnt10a* mRNA in nipple-proximal zones and with *Wnt6* and non-canonical *Wnt5a* mRNA in the TEB-distal regions. Intriguingly, Gpr125 and Wnt5a exert the opposite effects on rates of ductal elongation: loss of Gpr125 delays whereas loss of Wnt5a accelerates ductal elongation^59^, providing genetic evidence for pathway antagonism in this process. Sox11 also impinges on Wnt signaling, amplifying canonical Wnt signaling, but also transactivating Wnt5a^61,62^. Both Gpr125 and Sox11 regulate the asymmetric distribution of non-canonical WNT/Planar cell polarity protein, Vangl2^5,62^

A role for Gpr125+ cell-types in cell migration has considerable clinical significance in the setting of breast cancer, a disease where dissemination and metastasis are the primary cause of death. Our in silico analyses indicate that high levels of Gpr125 are associated with particularly poor survival within basal breast cancer, a subtype with a great unmet need for markers that can parse the 30% of patients that relapse and die within two years from the 70% with more indolent tumors. Studies using patient samples will now be needed to assess the prognostic utility of Gpr125 in this regard. In mouse models, Gpr125 mRNA is elevated and Gpr125+ cells are greatly expanded and massed at the invasive margins of MMTV-Wnt1 tumors that arise with very short latency^45^. This Gpr125+ cancer cell-type shares several features with Gpr125 progenitors found the multilayered tips of the embryonic mammary tree and pubertal TEB suggesting its pathological contribution to reduced latency may relate to an inherent genetic program governing invasive behavior of embryonic and pubertal progenitors. Sox11 is similarly elevated within nuclei at the pushing margins of mouse mammary tumors, and also when present at high levels correlate with increased metastasis and poor overall survival in human breast cancers^39,41,55,63^. The opposite is found for Wnt5a, where loss is associated with an increased risk of metastasis and a worse prognosis^59,64^. Thus, going forward it will be important to decipher intersections among these co-expressed genes to illuminate cell migratory processes in embryogenesis and their links to tumorigenesis.

## Methods

### Ethics statement

All studies involving animals received ethical approval by the Institutional Animal Care and Use Committee (IACUC) at NYU School of Medicine under protocols 202000144 and TR201900137/IA16-00513. All mice were housed in individually ventilated cages under a 12:12 h light–dark cycle with water and food available ad libitum and monitored daily in an ALAC accredited facility, and euthanized by CO_2_ anesthesia followed by cervical dislocation. Tumors induced in transgenic breast cancer models were not allowed to exceed 2 cM in diameter or 5% of body weight in accordance with IACUC guidelines.

### Mice

Males and females of the following strains were maintained as breeding stock. Females only were used in all analyses.

*Adgra3*^*cre*^ mice were constructed by Ingenious Technologies, Ronkonkoma, NY as follows. A cassette containing *CreER*^*T2*^ followed by a 3’ polyadenylation signal, harboring SV40-driven Neo flanked by FRT sites inserted in a central intron, was recombined into a bacterial artificial chromosome (BAC) to place *CreER*^*T2*^ under the control of the *Adgra3* promoter, excising 502 bp encompassing 221 bp of exon 1 and part of the following intron 1-2 of *Adgra3*. ES cells were selected for germline transmission by PCR, verified by southern analysis and sequencing then bred to B6.Cg-Tg(ACTFLPe)9205Dym/J (JAX: 005703) strain to remove Neo. These mice were crossed to FVB/N and B6.Cg-Gt(ROSA)26Sortm14(CAG-tdTomato)Hze/J (JAX: 007914) and maintained thereafter on a mixed strain background. *Adgra3*^*cre/cre*^ mice have been made available through the Mutant Mouse Resource and Research Center (MMRRC) Stock No. 068344 (https://www.mmrrc.org/catalog/sds.php?mmrrc_id=68344). Adgra3cre/+ were crossed to B6.Cg-Gt(ROSA)26Sortm14(CAG-tdTomato)Hze/J (JAX:007914) reporter strain for lineage tracing experiments. Tracing was activated at 5 weeks as well as during pregnancy P14.5 and P19.5 and tissue were harvested from mice 48 hrs later and at developmental time points 7, 8, 24, and 36 weeks, P15, P20.5, L6. For histological analysis, Adgra3^*cre/+*^ and Adgra3^cre/cre^ tissue were harvested at 6 weeks, P12.5, and involution days 3, 11, and 21.

*Adgra3*^*lz/+*^ mice^4^, kindly provided by Regeneron, were rederived and backcrossed onto an FVBN background. This strain was generated using VelociGene methods^65^ to modify a bacterial artificial chromosome (BAC) clone carrying the mouse Adgra3 gene by replacement of sequence encompassing exons 16–19 with *lacZ* to produce expression of fusion protein comprising the N-terminal extracellular domain, the first transmembrane domain, and part of the first intracellular loop of Gpr125 fused to β-galactosidase (Fig. 1a). Their mammary tissue was harvested during puberty (5–7 weeks of age) for flow cytometry, RNAseq, ductal elongation, and hormonal deprivation/supplementation studies. For X-Gal localization studies, tissue was harvested from nulliparous mice at day 2, day 18, weeks 3, 4, 6, 8, 12, 26, and 36, during early, mid and late pregnancy (P12, P13.5, P16.5, P18.5), lactation, involution days 1, 3, 5,10, 21 and from multiparous mice after 2 cycles of pregnancy and lactation (L6) and involution and from embryos at E14, E15, and E18.5.

MMTV-Wnt1 mice (FVB.Cg-Tg(Wnt1)1Hev/J Jax Strain #002934) were crossed to the above strains. Hyperplasia was analyzed at 7–8 weeks and tumors were collected before exceeding 2 cM in diameter or 5% of body weight in accordance with IACUC guidelines. These tissues were analyzed for Adgra3-β-gal expression, histology, and flow cytometry

Lgr5-EGFP-IRES-CreER (B6.129P2-Lgr5tm1(cre/ERT2)Cle/J Jax Strain #008875) and sSHIP-EGFP (B6.Cg-Tg(Inpp5d-EGFP)DLrr/CprJ Jax Strain #024808) mice were backcrossed onto on an FVBN background then to *Adgra3*^*lz/+*^ for immunolocalization and FACS experiments. Prepubertal 3-week old homozygous CrTac:NCR-Foxn1^nu^(NCRNU) (Taconic) females were used as hosts for mammary cell transplantation assays.

### Genotyping

All mice were analyzed using a standard cycle of 1 min each 94 °C, 58 °C, 72 °C for 30 cycles on a Perkin Elmer (Waltham MA) DNA Thermal Cycler Machine and the PCR products were visualized on a 2% agarose gel containing 0.2μ g/mL ethidium bromide (Sigma-Aldrich, St. Louis, MO). Oligonucleotide primers: Adgra3-cre-F 5′-TAA AGA TAT CTC ACG TAC TGA CGG TG-3′ and Adgra3-cre-R 5′-TCT CTG ACC AGA GTC ATC CTT AGC-3′, Adgra3-wt-F 5’-ACG CTG CCC AAC CGC A-3′ and Adgra3-wt-R 5′-AAA GCA GGG ATGGCA TGG GAC G-3′; Adgra3-lacZ-F 5′-ATG TTA GCT TAA ATG GAC TGT C-3′, Adgra3-lacZ-R 5’-GTC TGT CCT AGC TTC CTC ACT G-3′; Adgra3-wt-F 5’-AGA TGC ACC AAG GAA GCC AG-3′, Adgra3-wt-R 5′-ATA AGT AAA TCC CAA AGC TCA C-3′, MMTV-Wnt1-F 5′-GGA CTT GCT TCT CTT CTC ATA GCC-3′, MMTV-Wnt1-R 5’-CCA CAC AGG CAT AGA GTG TCT GC-3′. MMTV-Wnt1, sSHIP-EGFP and Lgr5-EGFP were genotyped according to their respective Jax Lab protocols.

### RNA isolation and qRT-PCR analysis

Inguinal mammary glands were harvested, snap-frozen in liquid nitrogen, and homogenized in 1 ml of TRIReagent (Life Technologies) using a hand-held tissue homogenizer (Kinematica, Lucerne, Switzerland), then mixed with 200 μl of chloroform and centrifuged at 14,000 × *g* for 15 min to eliminate protein debris. The upper aqueous phase was mixed with an equal volume of 70% ethanol and passed through a Qiagen RNeasy mini spin column by 15 s centrifugation at 8000 × *g* at room temperature (RT). Total RNA bound to the column filters was washed in 350 μl of ethanol-containing buffer (RW1 buffer; Qiagen, Valencia, CA, USA) to remove contaminants and incubated in 10 μl of RNase-free DNase I enzyme (273 Kunitz units; Qiagen) for 15 min at RT to ensure digestion of any residual genomic DNA fragments. The columns were washed according to the manufacturer’s instructions in ethanol-containing buffers (RW1 and RPE buffers; Qiagen). Total RNA was eluted in 50 μl of RNase-free water, and its concentration was determined by Nanodrop measurement. Reverse transcription was performed using 2 μl of RNA (10 ng/μl) from tissue using the QuantiTect Probe RT-PCR Kit (Qiagen; catalog number 204443). Real-time analysis was performed using the ThermoFisher Scientific Taqman Gene Expression Assay ID Mm01211377_m1 for mouse *Adgra3* and mouse *β2-microglobulin* (Mm00437762_m1) as the reference assay. Realtime analysis was performed in the Applied BiosystemsViiA™ 7. Total *Adgra3* mRNA levels were normalized to those of mouse β2 microglobulin and plotted as levels relative to tissue from males.

### Lineage tracing

Expression of the lineage reporter harbored by *Adgra3-CreER*^*T2*^;*Rosa26R-lox.STOP.lox-tdTomato* (*tdT*)was initiated by delivering tamoxifen (Tam). For tracing at mid-puberty Tam was delivered IP at low dose: 1.5 mg, and high dose: 5–15 mg (delivered in 2.5 mg aliquots every other day). For tracing during pregnancy and embryogenesis 2 doses of 2.5 mg Tam were given by oral gavage to *Adgar3*^*cre/cre*^ pregnant dams over a 24 h time period. Pups were delivered at E19.5-E20.5 by caesarian section to avoid Tam-induced problems with delivery and fostered by SWR/J mice. No fluorescence was observed in non-induced mice. Specific details of the timing of Tam delivery, age of mice, and intervals for harvesting of tissue are provided in each figure.

### Tissue clearing and 3-D imaging

Mammary glands were excised and fixed O/N in 4% paraformaldehyde (PFA) (Sigma-Aldrich) then processed using a CUBIC protocol optimized for mammary gland^25,66^. Tissue was incubated at 37 °C in CUBIC Reagent 1 A (10 wt% Triton, 5 wt% N,N,N’,N’-tetrakis (2-HP) ethylenediamine, 10 wt% Urea, NaCl 25 mM) clearing solution for 4 days, rinsed 3× in phosphate-buffered saline (PBS), then incubated at 4 °C for 4 days in primary antibodies diluted in PBS with Triton (PBST) containing 10% serum, rinsed again, then incubated at 4 °C in secondary antibody for 2 days, rinsed 3×, then cleared in CUBIC Reagent 2 (50 w/v% Sucrose, 25 w/v% Urea, 10 w/v% Triethanolamine, 0.1 w/v% Triton) at 37 °C for 24 h. Primary rabbit antibodies: anti-K5 (Covance, PRB160P, 1:100); anti-E-cadherin (Cell Signaling, 3195 S, 1:100); anti-p63 (Abcam, ab124762,1:100); anti-K14 (Abcam, Ab181595 1:100); and rat anti-K8 (Developmental Studies Hybridoma Bank, TROMA-I, 1:50); mouse anti-SMA (Dako, M0851, 1:100). Alexa Fluor-conjugated secondary antibodies (Thermo Fisher Scientific 1:500): goat anti-mouse 647 (A21237); goat anti-rat 647 (A21247); goat anti-rabbit 647 (A21245). Cleared mammary tissues were imaged using a Zeiss 880 Laser Scanning inverted confocal microscope with 10X, 20X air Plan-Apochromat N.A. 0.8 M27 objective lenses. Confocal images were displayed with ZEN2 software. This was followed by visualization and analysis in FIJI/Image J v.2.0.0 and Imaris v.9.5.

### X-gal staining

Embryos and mammary glands were fixed in 4% PFA at RT for 30-60 min, rinsed 3X in X-gal rinse buffer (2 mM MgCl_2_, 0.1% Sodium deoxycholate, and 0.2% NP-40 in PBS) at RT, then incubated in X-gal staining solution (50 mg/ml 5-bromo-4-chloro-3-indolyl-β Dgalactopyranoside) in rinse buffer containing 5 mM potassium ferricyanide, 5 mM potassium ferrocyanide) (Applichem, Cheshire, CT) at RT O/N. After staining, glands were rinsed in PBS, post-fixed in 4% PFA O/N then prepared for whole-mount analysis or processed for paraffin embedding, sectioning, and histological analysis.

### Mammary gland whole mounts

X-gal stained whole mounts were post-fixed in 4% PFA, washed twice with 1X PBS, dehydrated through an increasing ethanol gradient, cleared of lipids in Carnoy’s Fixative (60% Ethanol, 30% Chloroform, 10% Glacial Acetic Acid) for 2 h, and further cleared in Citrisolv (Fisher Scientific, Suwanne, GA) for 2 h. Glands were pressed flat between the slide and coverslip under a heavy weight for 30 min, and imaged on a Leica dissecting microscope Model WILD M3Z (Leica Microsystems, Bannockburn, IL) with an Optronics digital camera Model 60800 (Goleta, CA). The glands were then re-hydrated through a decreasing ethanol gradient and counterstained with Carmine alum (500 mL distilled water containing 1 g Carmine and 2.5 g aluminum potassium sulfate; Sigma Aldrich, St Louis, MO) diluted 1:4 in distilled water. Glands were once again dehydrated in ethanol, cleared in Carnoy’s Fixative and Citrisolv, and pressed flat before mounting under a coverslip with Cytoseal (VWR, West Chester PA) then re-photographed.

### Immunohistochemistry and immunofluorescence

Mammary glands were fixed with either 10% neutral buffered formalin or 4% PFA and embedded in paraffin. Tissue sections on slides were incubated at 60 °C oven for 1 h, rinsed in Citrisolv for 10 min, and rehydrated. Antigen retrieval was performed by microwaving at 900 watts for 30 min in 10 mM Citric Acid buffered to pH 6. From this point forward, the slides were washed thrice with 1× PBS between each step. For immunohistochemistry (IHC), endogenous peroxidase activity was quenched by treating slides with 3% Hydrogen Peroxide (Sigma Aldrich) for 15 min at RT. Slides were blocked with 20% normal goat serum for 30 min to reduce background signal. Primary antibodies were diluted in 2% bovine serum albumin (BSA, Sigma-Aldrich) in 1× PBS, and incubated at 4 °C O/N. Primary rabbit antibodies to: K14 (Covance PRB-155P 1:4000); Tcf1 (Cell Signaling 22035 1:100); Collagen (Rockland1:100), Fibronectin (Sigma F3648 1:100), Sox11 (Millipore ABN105, 1:100), Ki67 (Thermo Scientific 1:100), p63 (Abcam 4262 1:100), progesterone receptor(DAKO A0090 1:500), EGFP (Life Technologies A-11122 1:300) and mouse antibodies to K8 (Progen 65138 undiluted); E-cadherin (BD 610182 1:100); PCNA (Dako M0987 1:500), estrogen receptor (SRA 1010 StressGen 1:100), BrdU (Invitrogen 033900 1:500), p27(Thermo Scientific Ab1 MS-256 1:200) and goat antibodies to P-cadherin (R&D AF761 1:100). For immunofluorescence, Alexa Fluor-conjugated secondary antibodies from (Life technologies diluted 1:500): goat anti-mouse 647 (A21237); goat anti-rat 647 (A21247); goat anti-rabbit 647 (A21245), donkey anti-goat 555, (A21432) were applied for 30 min at RT. For IHC biotinylated secondary antibodies were diluted in 2% BSA/PBS for 1 h at RT, followed by HRP-conjugated Streptavidin (Vector Labs, Burlingame CA) for 30 min at RT. Colorimetric signal was developed using the DAB substrate (Vector Labs).

### Mammary epithelial cell preparation

All reagents were purchased from Stem Cell Technologies (Vancouver, British Columbia) unless otherwise noted. Mice were sacrificed with CO_2_ followed by cervical dislocation, and the 3rd, 4th, and 5th mammary glands were harvested and the lymph nodes excised. Glands were minced into a homogeneous slurry added to a 15 mL tube containing 1 mL Collagenase/Hyaluronidase and 9 mL Epicult-B Basal Medium and rotated for 6–8 h at 37 °C. The digests were then vortexed briefly and centrifuged at 450 × *g* for 10 min. The pelleted epithelial organoids were resuspended in 1 mL of 0.25% Trypsin-EDTA and incubated at 37 °C for 1 min, quenched with HBSS supplemented with 2% FBS (HF), and centrifuged at 450 × *g* for 6 min. The pellet was resuspended in 1 mL Dispase with 1 mg/mL DNase I (Roche, Indianapolis, IN) for 1 min, pelleted, and resuspended in NH4Cl red blood cell (RBC) lysis buffer for 1 min at RT. A single-cell suspension was made by filtering through a 40 µm mesh strainer (BD, East Rutherford, NJ) into 5 mL HF.

### Flow cytometry and cell sorting

To detect Gpr125-β-gal expression, cells were labeled with fluorescein di-V-galactoside (FDG) according to the manufacturer’s protocol as follows (Molecular Probes, Eugene, Oregon). MECs were prepared as described above from 3 to 4 mice/genotype. For FDG staining, cells were resuspended at 10^7^/mL in HBSS supplemented with 2% FBS, and the samples were pre-warmed at 37 °C for 10 min. FDG loading was performed by adding an equal volume of pre-warmed 2 mM FDG (diluted in distilled water) to the cell suspension for exactly 1 min at 37 °C, then immediately quenched by adding 2 mL ice-cold HF. The FDG-loaded cells were then centrifuged 1000 × *g* and stained with surface antibodies. The following antibodies were used to label cells for flow cytometry: biotinylated- TER119 (BD 553672,1:200), biotinylated-CD31 (BD 558737,1:200), biotinylated-CD45 (BD 553077,1:200), biotinylated-CD140a (eBioscience 12-1401-80,1:200), CD24-PE (BD 553262,1:400), CD49f-PerCP-Cy5.5 (Biolegend 313617,1:200,), CD49f-PE-Cy7(BD 313621 1:200), CD24-FITC (BD553261,1:100), CD49f-PE (BD 313611 1:100), Streptavidin-AlexaFluor647 (Molecular Probes S21374,1:600), CD61-APC (Caltag,1:200), Sca1-PE-Cy7 (eBioscience 25-5981-81,1:600), CD29-Pacific Blue (Biolegend 102224,1:200). Cells were incubated with conjugated antibodies diluted in HF, for 30 min on ice in a dark container, washed with 2 mL of HF, and resuspended in 250 μL HF for analysis. Cell viability was assessed by adding 4′,6-Diamidino-2-phenylindole (DAPI, Sigma-Aldrich) to the final suspension at a concentration of 1 μg/mL. Data collection for flow cytometry was done on a Beckton-Dickinson (East Rutherford, NJ) LSRII analyser. Analyses were done using FlowJo software version 9. Gating strategy is reported in Supplementary Fig. 8. For cell sorting, cells were stained as described above, and FDG+/CD49f^hi^ and FDG-/CD49f^hi^ were sorted using FACSAria IIu and processed for mRNA extraction.

### Transplantation assay

MECs were prepared as described above. Cleared fat pads from 3-week-old female *FoxN1*^*nu*^ mice were transplanted with 10^5^ MECs in 50/50 Matrigel/minimal medium obtained from a #4 inguinal gland from (*n* = 4) 7-week-old *Adgra3-CreER*^*T2*^*;Rosa26*^*tdT*^ mice that were injected at puberty with Tam to assess the potency of Gpr125 parental cells. Mammary outgrowths of recipient glands (*n* = 4) were analyzed 3 weeks later by 3D imaging to assess the potency of Adgra3 cells. The remaining #4 inguinal gland of the donor *Adgra3-CreER*^*T2*^*; Rosa26*^*tdT*^ mice (*n* = 4) were harvested and analyzed as controls of this experiment.

#### Ovariectomy and hormonal supplementation

Two pairs of pre-pubertal and adult *Adgra3*^*+/+*^ and *Adgra3*^*lz/+*^ mice were bilaterally ovariectomized. After 10 days, pubertal mice received vehicle or estradiol at a concentration of 200 nM in their drinking water and adult mice were injected subcutaneously daily for 14 days with vehicle or 10 μg 17β-estradiol and 1 mg progesterone in 100 μl sesame oil (10.1210/en.2016-1480). Twelve hours before sacrifice, each animal received an IP injection of EdU (0.25 mg in 100-μL saline, ip; Life Technologies). Mammary glands were harvested and analyzed by X-gal staining. EdU incorporation was visualized using Click-iT™ EdU Cell Proliferation Kit for Imaging, Alexa Fluor™ 647 dye following the manufacturer’s instructions (Invitrogen).

#### Single-cell RNA seq analysis

The single-cell RNA seq datasets from embryonic (GSE109711)^30^, pubertal GSM2759554 and GSM2759555) and adult MECs (GSM2510617 and GSM2510616)^28^ were obtained from Gene Expression Omnibus (GEO) in the NCBI data repository and analyzed using iCellR R package (v1.5.5) (https://CRAN.R-project.org/package=iCellR)^43^. For each dataset quality control number of genes, UMIs, and the proportion of mitochondrial genes for each cell was calculated. Cells with low number of covered genes (gene-count < 200) and high mitochondrial counts (mt-genes > 0.08) were filtered out. This resulted in a total of 378 embryonic, 10,828 pubertal, and 7146 adult epithelial cells available for downstream analysis. Matrices were normalized based on their ranked geometric library size factor (ranked glsf). A general statistical analysis was then performed to calculate gene dispersion, base mean, and cell coverage in order to build a gene model for performing principal component analysis (PCA). Genes with high coverage and dispersion (dispersion > 1.5) were chosen to perform PCA (2000 genes). Clustering was performed on the principal components with high standard deviation (top 10 PCs) with dimensionality reduction by T-distributed stochastic neighbor embedding (t-SNE) and Uniform manifold approximation and projection (UMAP). Next, marker genes for each cluster were determined based on fold-change and adjusted *p*-value (*t*-test), and average gene expression for each cluster was calculated. Marker genes were identified for each cluster and visualized on heat maps, bar plots, and box plots. The marker genes were then used to determine basal and luminal epithelial cell types. For the pubertal dataset re-clustering of the three basal clusters were done to detect subpopulations. PCA was performed and identified clusters were visualized comparing *t*-SNE, UMAP, and K-nearest-neighbor-based Network graph drawing Layout (KNetL map). The zoom on KNetL map was set to 100 to detect sub-populations in cell communities using a force-based network layout to assign attractive (analogous to spring force) and repulsive forces (usually described as analogous to the forces in atomic particles) to separate all pairs of nodes of the network layout. Clustering using PhenoGraph^67^ was then performed based on the KNetL map dimensions. Marker genes were visualized on heat maps, bar plots, and box plots to characterize *Adgra3* expressing cells. The t-SNE plots of Fig. 2, panel D were generated using available data of whole mammary gland single cells from Tabula Muris (https://tabula-muris.ds.czhub.org/). Plots throughout the paper were generated using R Studio v 1.2.5019.

#### Transcriptome (RNA-seq) and data analysis

RNA seq of FACS-sorted Gpr125+ with Gpr125‒ cells isolated from TEB-distal and nipple-proximal regions of pubertal *Adgra3*^*lz/+*^ mammary glands was performed at the NYU School of Medicine Genome Technology Core. Total RNA was extracted from centrifugated cell pellets using RNeasy Plus MiniKit (cat, #74136, Qiagen) according to the manufacturer’s instructions. RNA quantity and quality were determined using Bioanalyzer RNA Quality and Quantity Assay NANO (Agilent Technologies, Santa Cruz, CA). RNA-seq library was prepared with the Low input Clontech SMART-Seq HT. The sequencing was performed using the Illumina NovaSeq 6000. For data analysis, all the sequencing reads were mapped to the reference genome (mm10) using the STAR aligner (v2.5.0c)^68^. Alignments were guided by a Gene Transfer Format (GTF) file. The mean read insert sizes and their standard deviations were calculated using Picard tools (v.1.126) (http://broadinstitute.github.io/picard). The Read Per Million (RPM) normalized BigWig files were generated using BEDTools (v2.17.0)^69^ and bedGraphToBigWig tool (v4). HTSeq (v0.6.0)^70^ was used to generate the read count tables. R (v.3.5.1; http://www.R-project.org/) and the DESeq2 package (v.1.10.0)^71^ were used to normalize samples based on their library size factors and to perform differential expression (DE) analysis among the different sample groups. To compare the level of similarity among the samples and their replicates, we used two methods: principal component analysis and Euclidean distance-based sample clustering. DE analysis identified 682 differently expressed genes comparing TEB-distal Gpr125^+^ vs Gpr125^‒^ cells and 317 genes comparing nipple-proximal Gpr125^+^ vs Gpr125^‒^ cells (2-fold change, FDR < 0.1). The Venn’s diagram of the two comparisons was derived using https://bioinfogp.cnb.csic.es/tools/venny/ and yielded 140 overlapping genes, which were further evaluated by GSEA and gene ontology (GO) using the ToppFun query tool of the ToppGene suite (https://toppgene.cchmc.org/)^72^. A preranked analysis was performed using log2 fold change as the ranking metric. Only gene sets with an FDR of <0.1 were included and plotted as -log10(FDR B&H) in bar diagrams. Differentially expressed genes between TEB-distal Gpr125^+^ and nipple-proximal Gpr125^+^ cells were visualized by Volcano plot (twofold change, p(adj) < 0.05), queried using ToppFun, and the relevant processes and functions related to these genes were plotted as bar graphs.

### Microarray analysis

*GPR125* mRNA expression in murine and human samples of breast cancer was obtained by using microarray data GSE3165 (https://www.ncbi.nlm.nih.gov/geo/). Analysis was conducted using GEO2R. Version info: R 3.2.3, Biobase 2.30.0, GEOquery 2.40.0, limma 3.26.8.

Analysis of *GPR125* in human breast cancer was carried out using kmplotter https://kmplot.com/analysis/index.php?p=service&cancer=breast with criteria: Gpr125 affy ID 210473_s_at, Auto select best cutoff, excluding biased arrays and selecting for basal-type breast cancer RFS: *N* = 618; DMSF: *N* = 232 and using BreastMark: http://glados.ucd.ie/BreastMark/mRNA_custom.html DFS, median cut off was selected for ssp2003 basal-type (*N* = 318)^73^ or ssp2006 basal-type (*N* = 366)^74^ datasets.

### Statistics and reproducibility

Statistical significance was determined using GraphPad Prism software v.9.2.0. Normal distribution of data was assessed using Shapiro-Wilk normality tests. Unpaired Student’s *t*-test or Mann–Whitney tests were performed as reported in the figure legends and “Support data” file. Data are always expressed as mean ± SEM. *p*-values. In all experiments *n* =  number of biological replicates as indicated in the Fig. legends.

### Reporting summary

Further information on research design is available in the Nature Research Reporting Summary linked to this article.

## Supplementary information

Supplementary Information

Description of Additional Supplementary Files

Supplementary Movie 1

Supplementary Movie 2

Reporting Summary

## Data Availability

The authors declare that all data supporting the findings of this study are available within the article and its supplementary information files or from the corresponding authors upon request. All RNAseq data used in this study have been deposited in the Gene Expression Omnibus (GEO) database accession number GSE190180 and are publicly available. Previously published scRNAseq data that were re-analyzed here are available under the following accession codes GSE109711: GSM2759554: GSM2759555: GSM2510617: GSM2510616: The mm10 reference genome: http://ftp.ensembl.org/pub/release102/fasta/mus_musculus/dna/ Microarray data: GSE3165 (https://www.ncbi.nlm.nih.gov/geo/) The cBioPortal platform was used to access human breast cancer data derived from TCGA Pancancer and Metabric https://www.cbioportal.org/study/summary?id=brca_tcga_pan_can_atlas_2018https://www.cbioportal.org/study/summary?id=brca_metabric All source data related to graphs within the figures is provided with this paper as an excel file titled “Source Data”. *Adgra3*^*cre/cre*^ mice generated in this study are available via the Mutant Mouse Resource and Research Center (MMRRC) Stock No. 068344. Source data are provided with this paper.

## Source data

Source Data

## Code availability

No in house codes/pipelines were used. All the tools and versions listed in the reporting summary are publicly available.
