# Supplementary material for: Gpr125 is a unifying hallmark of multiple mammary progenitors coupled to tumor latency
Source: Nat Commun. 2022 Mar 17;13:1421. doi: 10.1038/s41467-022-28937-x (PMC8931046; doi:10.1038/s41467-022-28937-x)
Supplement: Supplementary file 1 — Supplementary Information [file 41467_2022_28937_MOESM1_ESM.pdf]

Supplementary Figure1

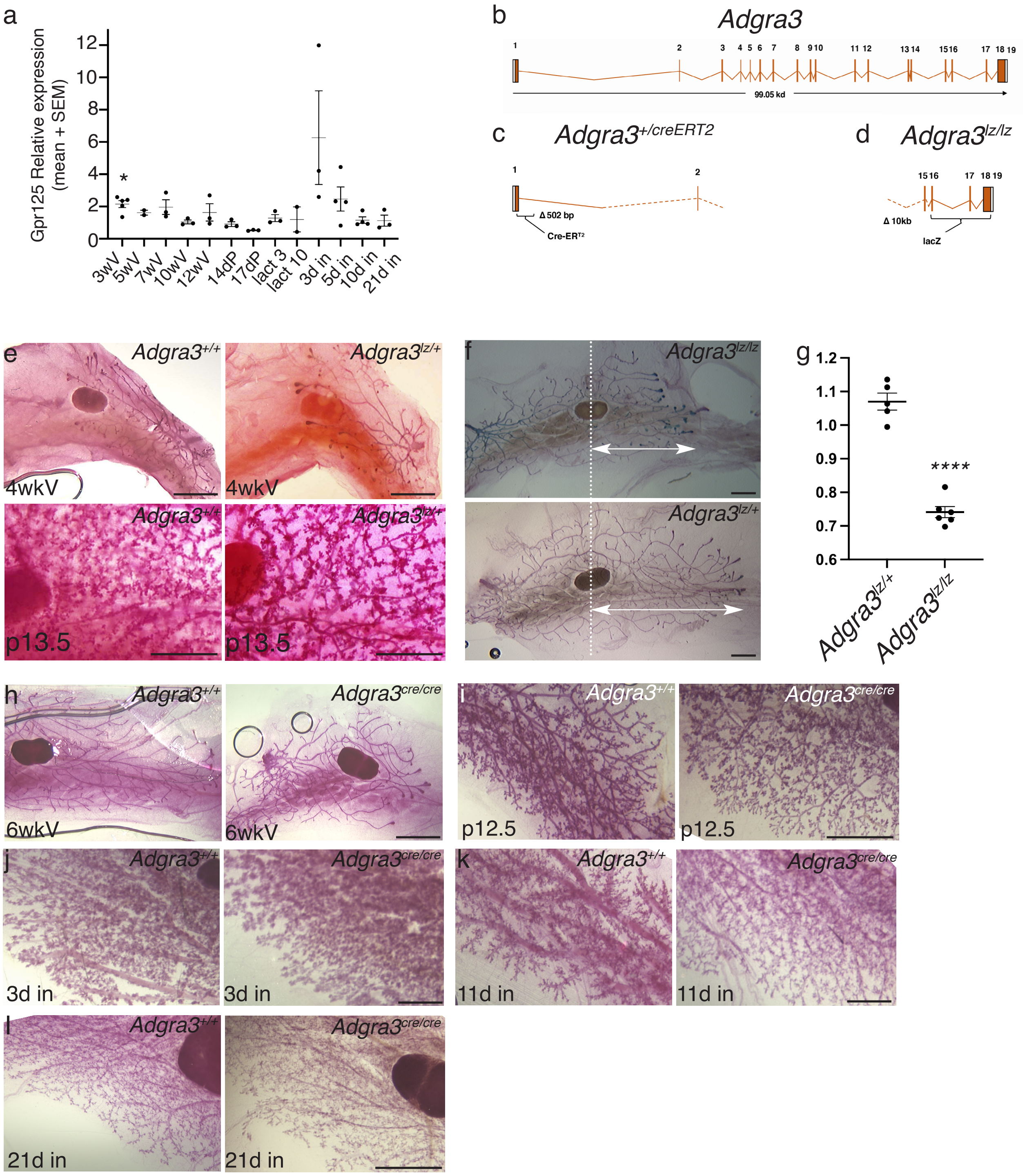

Supplementary Fig.1. *Adgra3*$^{lz/+}$ and *Adgra3*$^{cre/+}$ mouse strains and mammary phenotypes. a) Scatter dot plot of *Adgra3* relative mRNA levels at different stages of mammary development. Each dot represents the mean of 4 technical replicates on one individual mouse normalized to male mammary glands; Bars indicate the mean values +/- SEM of the number of mice (n) indicated at each stage (see support data file). wV = weeks of age virgin nulliparous mice, dP = days of pregnancy; lact=lactation; d in=involution day. p=0.0121*; Mann Whitney test two-tailed. b) Schematic of *Adgra3* gene. c) *Adgra3*$^{cre/+}$ mice were generated by replacement of 502 bp after the first codon with a cassette containing c*reER*^{T2}. d) *Adgra3*$^{lz/+}$ mice[1] were generated by deletion of 10 kb sequence downstream of the first TM and replacement by *lacZ* inserted in frame using Regeneron Velocigene technology. e) Carmine-stained mammary whole mounts from 4-week-old female virgin (upper panel) and pregnant p13.5 (bottom panel) *Adgra3*$^{lz/+}$ and *Adgra3*$^{lz/lz}$ mice. f) Ductal elongation in mammary wholemounts from pubertal *Adgra3*$^{lz/lz}$ females compared to littermates. g) Quantitation of ductal elongation with respect to the midpoint of the inguinal lymph node marked by the dashed line in f show impairment in *Adgra3*$^{lz/lz}$ (n=5) compared to *Adgra3*$^{lz/+}$ (n=6); p<0.0001****. Data are presented as mean values +/- SEM using the unpaired two-tailed t-test (see support data file). h-j) Carmine-stained mammary whole mounts from *Adgra3*$^{+/+}$ and *Adgra3*$^{cre/cre}$ mice at 6-week-old nulliparous (h), pregnancy day 12 (p12.5) (i); involution day 3 (3d in) (j), day 11(11d in) (k), day 21 (21d in) (l). (n= glands from 3 mice/stage). Scale bar = 2mm.

Supplementary Figure 2

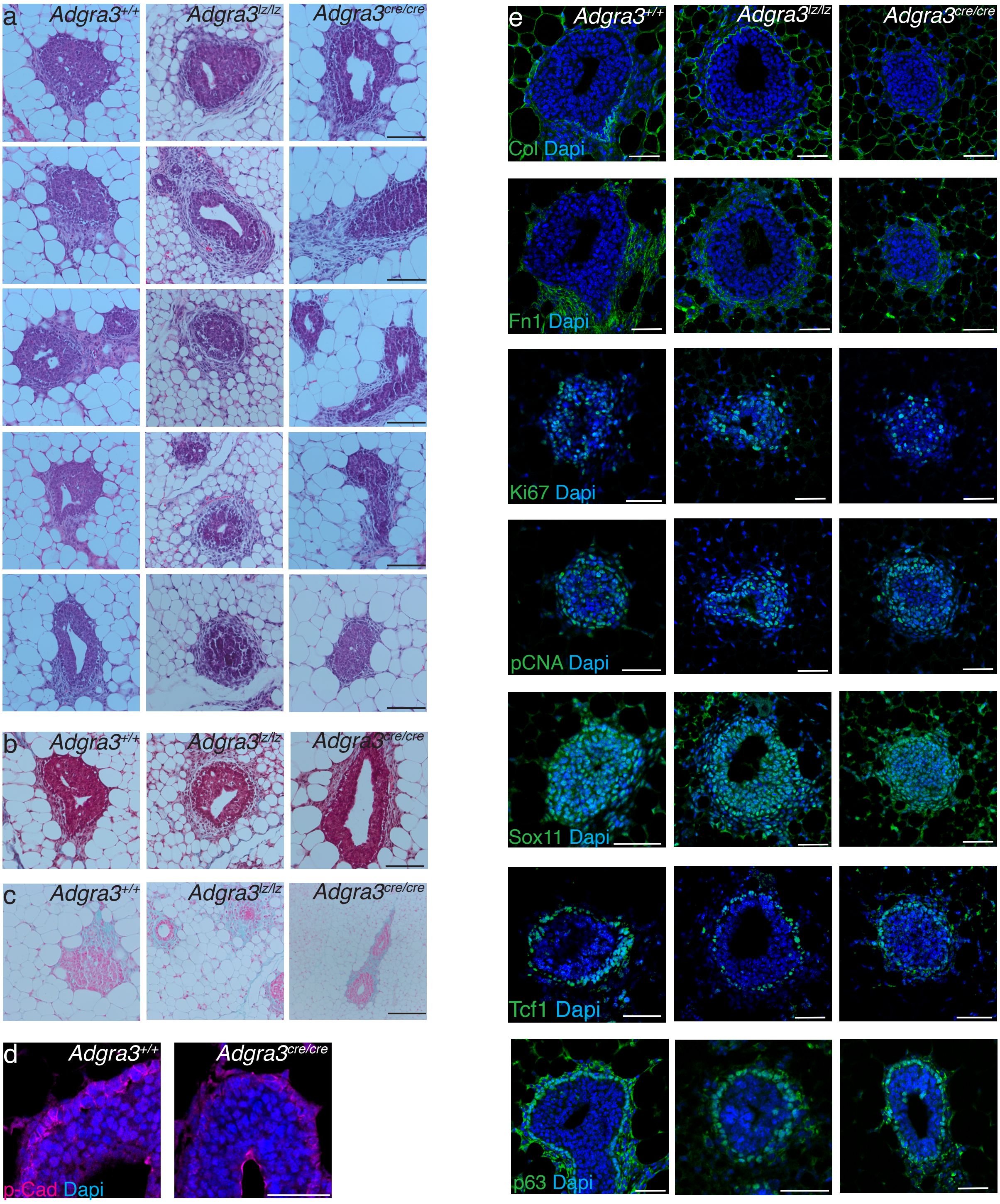

Supplementary Fig. 2. Comparison of terminal end buds from *Adgra3*$^{+/+}$, *Adgra3*$^{lz/lz}$ and *Adgra3*$^{cre/cre}$ mice

Terminal end buds from *Adgra3*$^{+/+}$ *Adgra3*$^{lz/lz}$ and *Adgra3*$^{cre/cre}$ mice (a) stained with H/E; (b) Masson's Trichrome to detect collagen; (c) Alcian Blue to detect hyaluronan; (d) anti-P-cadherin (P-cad) (magenta) to detect points of cell-cell contact between cap cells; (e) antibodies as indicated against ECM components: collagen (Col), Fibronectin (Fn1), markers of proliferation: Ki67 and PCNA and transcriptional regulators: Sox11, Tcf1, p63. All sections were counterstained with DAPI (blue, nuclear staining). Scale bar = 50μm. (n=3 mice).

Supplementary Figure 3

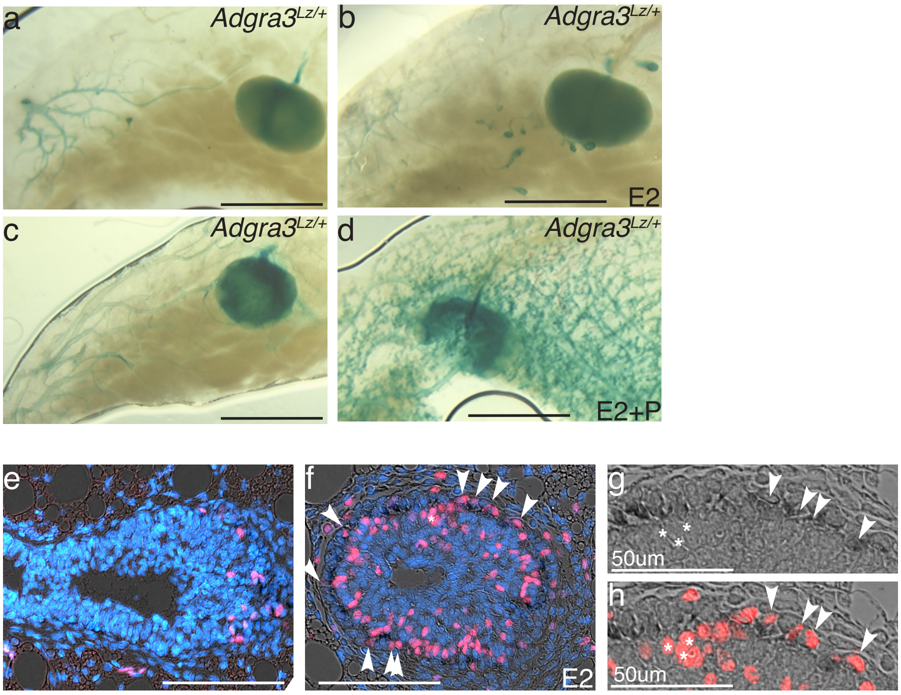

Supplementary Fig.3. Effect of hormonal deprivation and replacement on Gpr125 expressing basal cells of TEBs.
a,b) X-gal (blue) stained mammary gland whole mounts from female Adgra3$^{lz/+}$ mice ovariectomized at the onset of puberty, 3-weeks-old (a,b), or as adults (c,d) then treated with vehicle (a,c), estradiol (b), or estradiol and progesterone (d). Scale bar = 2mm. e,f) Representative compound images of sections of ductal tips from pubertal Adgra3$^{lz/+}$ mice untreated (e) and treated with estradiol (f) stained with X-gal (dark grey), and with click-it detection of EdU incorporation (pink). All nuclei stained with DAPI (blue). Scale bar = 100μm. g,h) Higher magnification reveals cap cells showing X-gal detection of Gpr-lacZ expression (arrowheads dark grey stain), as well as adjacent body cells (asterisks), both incorporate EdU in pink. Scale bar = 50μm. (n= 2 pairs of mice/condition).

Supplementary Figure 4

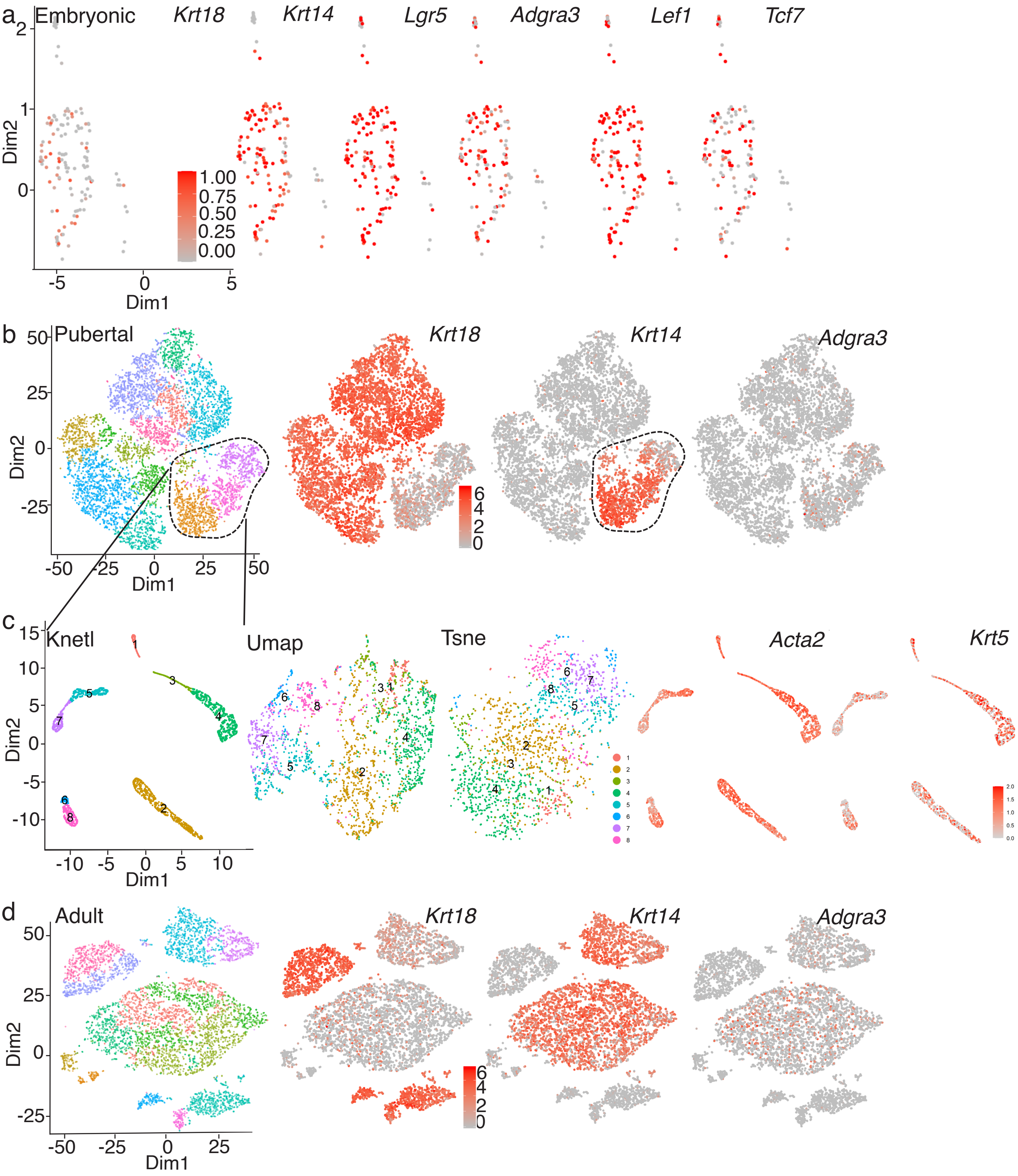

Supplementary Fig. 4. Gpr125 cells from pubertal and adult mice are confined to the basal cluster.

scRNA-seq datasets were analyzed from embryonic[2], as well as pubertal and adult mammary glands[3] using the iCellR pipeline[4]. a) t-SNE plots of *Krt18, Krt14, Lgr5, Adgra3, Lef1 and Tcf7* in embryonic mammary cells. b) t-SNE plots of *Krt18*, *Krt14* and *Adgra3* in pubertal mammary cells show *Adgra3* expressing cells within the basal cluster. c) Clusters of the subset of pubertal basal cells (dotted circle) are shown in KNetL, UMAP and tSNE and expression levels of *Acta2* and *Krt5* are depicted in KNetL plots. KNetL provided the most robust cluster separation and was used to characterize *Adgra3* expressing cells (Fig. 2j). d) t-SNE plots of *Krt18*, *Krt14* and *Adgra3* in adult mammary cells.

Supplementary Figure 5

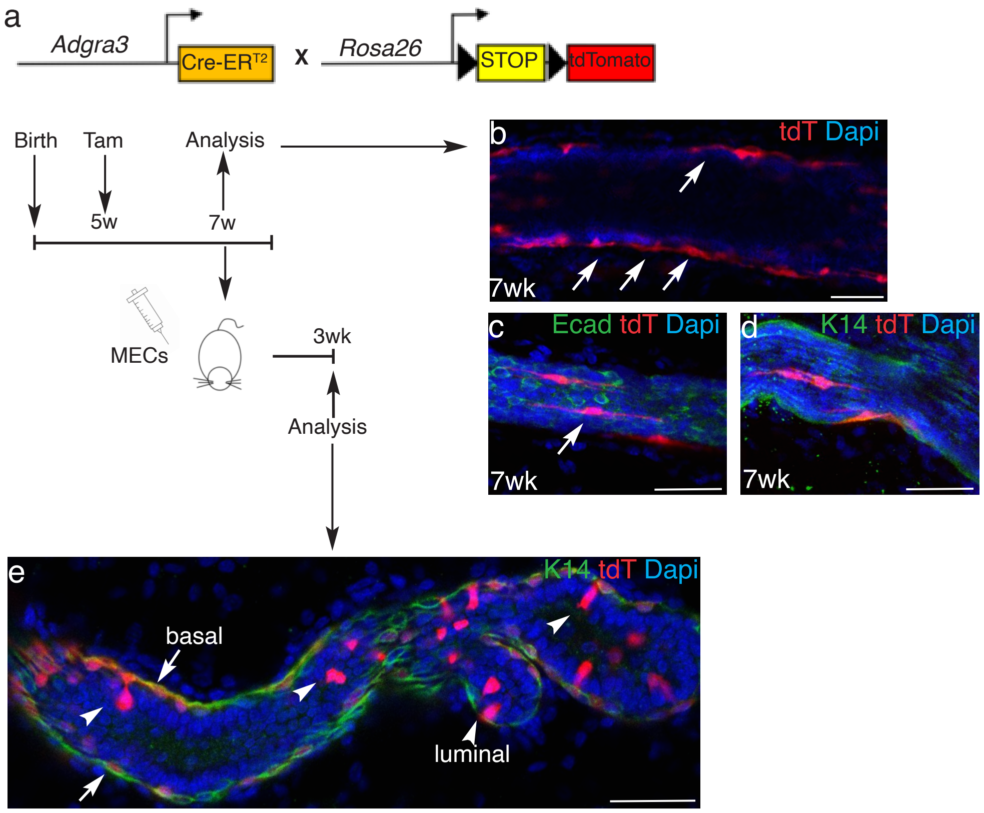

Supplementary Fig.5. Gpr125 cells generate bilineage progeny after transplantation.
a) Strategy showing activation of the *Rosa26*^{tdT} lineage reporter in *Adgra3*^{creERT2} expressing cells by Tam injection during early puberty in donor Gpr125 mice, followed by transplantation of cells derived from a #4 inguinal gland (n= 4 mice) into cleared mammary fat pads of *Foxn1* recipients (n=4 mice). Recipient glands were analyzed 3 weeks after transplantation and remaining donor glands at 7 weeks of age. b) Immunofluorescence 3D confocal imaging of endogenous glands left in the donor shows stripes of tdT+ (red) cells (white arrows) exclusively in the outer basal layer of ducts that c) lack luminal marker Ecad (green) but d) express basal marker K14 (green). Nuclei are stained with DAPI (blue). e) In contrast, a mixed lineage of cuboidal luminal cells (white arrow head) and spindle shaped basal tdT+ cells (white arrow) were found in ducts regenerated within the *Foxn1*^{nu} recipients. Scale bar = 50 μm.

Supplementary Figure 6

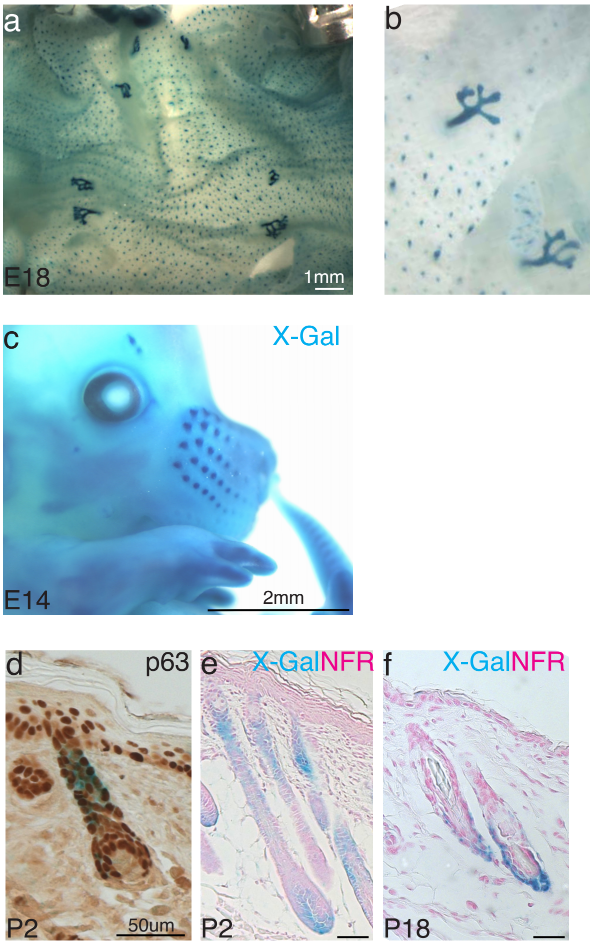

Supplementary Fig. 6. Gpr125 expression in other ectodermal appendages.
a, b) X-gal(blue) stained skin from E18 embryo shows Gpr125-β-gal expression in the five pairs of mammary trees and developing hair follicles (n=3 embryos). Scale bar = 1mm. c) Whole mount of E14 embryo showing robust Gpr125-β-gal expression in the whisker-pad. Scale bar = 2mm (n=3 litters of embryos). d-e) Sections showing Gpr125-β-gal expression concentrated in the bulge and bulb compartments of P2 hair follicles during anagen, (d) immunolocalized with p63 (brown), or (e) counterstained with NFR f) Gpr125-β-gal expression in the secondary germ of P18 hair follicles during telogen. Scale bar = 50 μm. (n=skins from 2 mice/stage).

Supplementary Figure 7

a

Mouse Tumor Model	Fold change
Wnt1-Early^Ex	3.17
p53null-Basal^Ex	1.76
Wnt1-Late^Ex	1.7
Class14^Ex	1.31
Class8^Ex	1.2
p53null-Luminal^Ex	0.95
Squamous-like^Ex	0.9
Neu^Ex	0.9
Erbb2-like^Ex	0.9
PyMT^Ex	0.8
Class3^Ex	0.78
Stat1^Ex	0.77
C3Tag^Ex	0.76
Myc^Ex	0.75
Normal^Ex	0.7
ClaudinLow^Ex	0.68
WapInt3^Ex	0.6

b

Human Breast Cancer subtype	Fold change
Human Basal-like	2.03
Human Normal-like	1.33
Human LumB	0.78
Human LumA	0.77
Human Her2-Enriched	0.75
Human Claudin-low	0.8

c

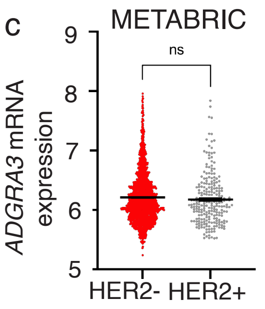

d

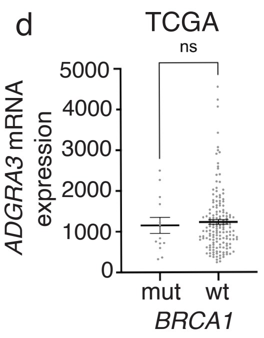

e

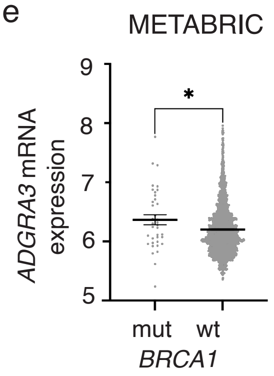

f

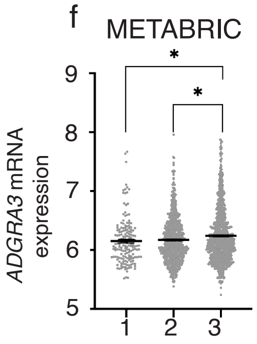

h

ADGRA3 expression	Cell line name	Tumor Subtype Classification
5.71	MDAMB436	Basal B
5.21	HCC1143	Basal A
5.15	HCC1187	Basal A
5.09	CAL51	Basal B
4.81	MDAMB361	HER2+ ER+
4.80	SUM159PT	Basal B
4.76	HCC70	Basal A
4.70	CAL148	LUM
4.60	HCC1569	Basal A
4.58	MDAMB231	Basal B
4.50	HMC18	Basal B
4.40	HCC1599	Basal A
4.31	HCC1954	Basal A
4.20	CAL851	Basal A
4.10	BT549	Basal B
4.03	HCC1500	Lum
4.02	BT20	Basal A
3.92	BT483	Lum
3.88	CAMA1	Lum
3.88	HS578T	Basal B
3.86	HCC1419	HER2 ampl
3.85	ZR751	Lum
3.84	CAL120	Basal B
3.82	EFM192A	HER2 ampl
3.81	SUM229PE	Basal
3.79	SUM149PT	Basal B
3.77	BT474	HER2 ampl
3.71	HCC1806	Basal A
3.68	MDAMB157	Basal A
3.67	HCC1395	Basal B
3.66	HDQP1	Basal A
3.57	SUM1315MO2	Basal
3.56	HCC2157	Basal A
3.51	MDAMB468	Basal A
3.37	MFM223	Lum
3.33	KPL1	Lum
3.16	T47D	Lum
3.15	MDAMB415	HER2 ampl
3.11	HCC1428	Lum
3.05	MCF 7	Lum
2.98	EFM19	Lum
2.92	SUM52PE	Lum

g

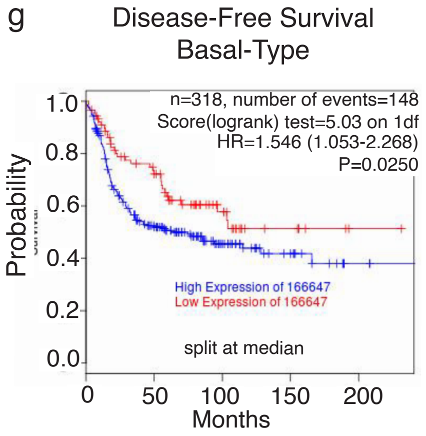

Supplementary Fig. 7. *Adgra3/ADGRA3* expression in murine and human breast cancer subtypes. a) *Adgra3* mRNA expression fold change values in mouse tumor models of breast cancer show highest expression in Wnt1-Early^{Ex}, p53null-Basal^{Ex} and Wnt1-Late^{Ex} tumors. b) *AGDRA3* expression fold changes, obtained by extracting microarray data available at the Gene Expression Omnibus under the series GSE3165 [5], are highest in human basal-like breast cancers. Scatter dot plots representing the mean +/- SEM of ADGRA3 expression show: c) no significant (ns) difference between HER2+ (n=236) and HER2- (n=1668) BC patients within the METABRIC dataset [6]; d,e) correlation with BRCA1 mutation (n=14) versus BRCA1 wt (n=158) within the TGCA cohort of basal BC (p=0.609 ns,not significant) (d) and the METABRIC cohort of all BC (e) (mut n=37,wt n=1867;p=0.0322*). f) correlation with higher histological grade in the METABRIC cohort (Grade 1 n= 165, Grade 2 n=740, Grade 3 n=927) p=0.033*. p-values were calculated using Mann Whitney test two tailed (see “Support data” file). g) Kaplan-Meyer curve generated by the BreastMark (http://glados.ucd.ie/BreastMark/) [7] algorithm for the ssp 2003/2006 dataset divided at the median expression level into low (red line) and high (blue line) groups indicates increased disease-free survival in BC patients with low *ADGRA3* expression levels score n=318 (logrank) test = 5.03 on 1 df, p=0.02495 with Hazard ratio HR = 1.546 (1.053 - 2.268). h) *ADGRA3* expression level (left column) in human breast cancer cells lines (middle column) with tumor subtype classification (right column) derived from the DepMap portal (https://depmap.org/portal/). Highest *AGDRA3* expression levels are more frequent in cell lines resembling basal tumor subtypes.

Supplementary Figure 8

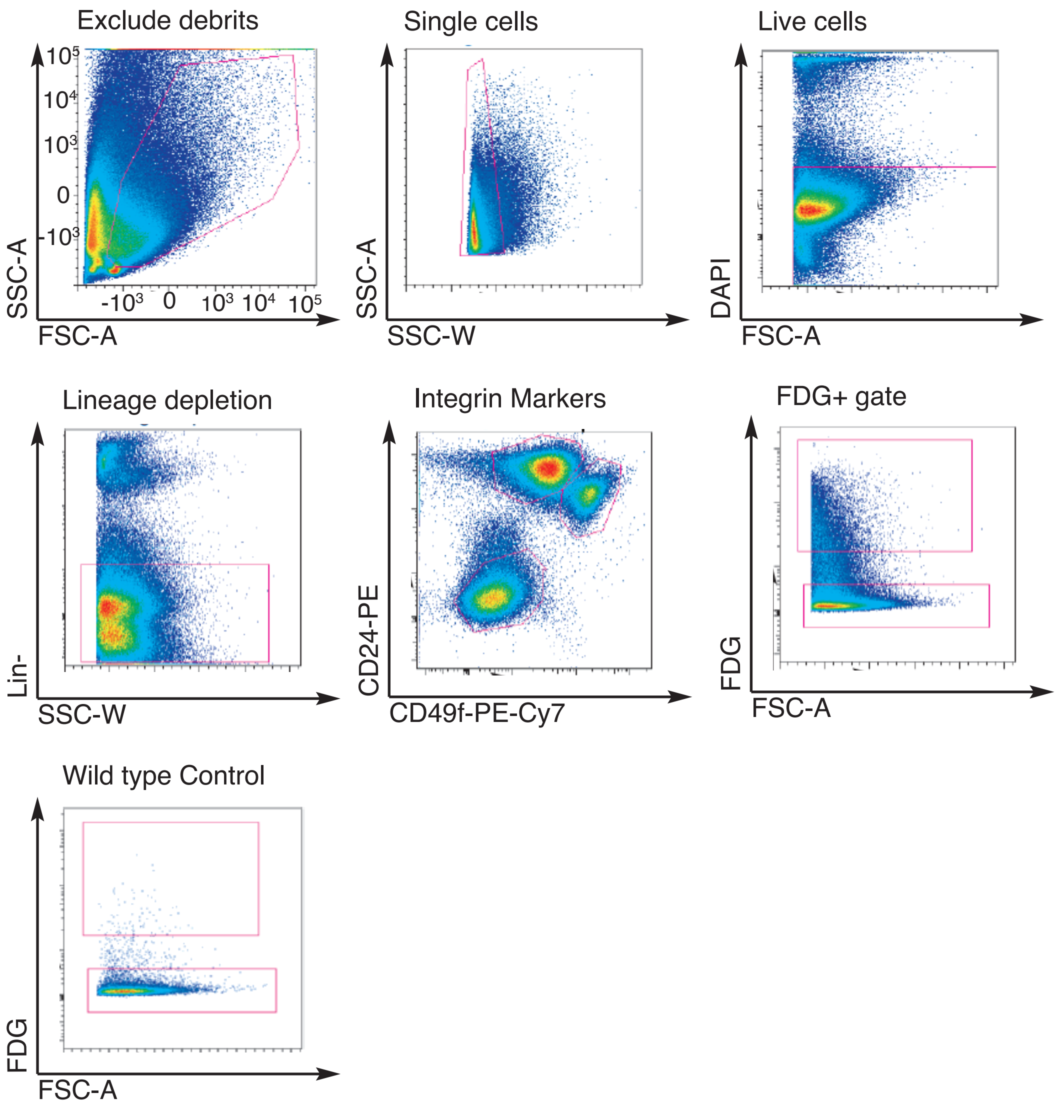

Supplementary Fig. 8 Gating Strategy for flow cytometry.
Exclusion of debris by forward scatter (FSC-A) and side scatter (SSC-A). Selection of single cells by width (SSC-W). Selection of live cells with DAPI. Exclusion of endothelial and hematopoietic lineages (Lin-). Typical profile of MECs from a 6-week-old mouse stained with surface markers for CD24-PE (Y-axis) and CD49f-PECy7 (X-axis). Positive signal for FDG in GPR+/LacZ cells, compared to background signal in wild-type control.

1 Seandel, M. *et al.* Generation of functional multipotent adult stem cells from GPR125+ germline progenitors. *Nature* **449**, 346-350, doi:10.1038/nature06129 (2007).
2 Wuidart, A. *et al.* Early lineage segregation of multipotent embryonic mammary gland progenitors. *Nat Cell Biol* **20**, 666-676, doi:10.1038/s41556-018-0095-2 (2018).
3 Pal, B. *et al.* Construction of developmental lineage relationships in the mouse mammary gland by single-cell RNA profiling. *Nat Commun* **8**, 1627, doi:10.1038/s41467-017-01560-x (2017).
4 Tang, K. H. *et al.* Combined Inhibition of SHP2 and CXCR1/2 Promotes Anti-Tumor T Cell Response in NSCLC. *Cancer Discov*, doi:10.1158/2159-8290.CD-21-0369 (2021).
5 Herschkowitz, J. I. *et al.* Identification of conserved gene expression features between murine mammary carcinoma models and human breast tumors. *Genome Biol* **8**, R76 (2007).
6 Curtis, C. *et al.* The genomic and transcriptomic architecture of 2,000 breast tumours reveals novel subgroups. *Nature* **486**, 346-352, doi:10.1038/nature10983 (2012).
7 Madden, S. F. *et al.* BreastMark: an integrated approach to mining publicly available transcriptomic datasets relating to breast cancer outcome. *Breast Cancer Res* **15**, R52, doi:10.1186/bcr3444 (2013).
