# Supplementary material for: Gpr125 is a unifying hallmark of multiple mammary progenitors coupled to tumor latency
Source: Nat Commun. 2022 Mar 17;13:1421. doi: 10.1038/s41467-022-28937-x (PMC8931046; doi:10.1038/s41467-022-28937-x)
Supplement: Supplementary file 2 — Description of Additional Supplementary Files [file 41467_2022_28937_MOESM2_ESM.pdf]

Description of Additional Supplementary Files

File Name: Supplementary Movie 1
Description: **tdT+ myoepithelial progeny of pubertal Gpr125 progenitors in lactating glands.** Video of 3D confocal imaging of tdT+ cells (red) in glands from Adgra3$^{cre/+;}$tdT mice treated with Tam during puberty. Glands were harvested at lactation day 6 (L6). Nuclei stained with DAPI (pale blue).

File Name: Supplementary Movie 2
Description: **tdT+ cells are at the invasive front of emerging branch from Adgra3- Wnt1 tumor.** Video of 3D confocal imaging of tdT+ cells (red) at the invasive front of tumor from MMTVWnt1; CreER^{T2};tdT mice. Mice were treated with Tam during puberty. Tumors were harvested ~15 weeks of age. Nuclei stained with DAPI (pale blue).
