# Supplementary material for: Gpr125 is a unifying hallmark of multiple mammary progenitors coupled to tumor latency
Source: Nat Commun. 2022 Mar 17;13:1421. doi: 10.1038/s41467-022-28937-x (PMC8931046; doi:10.1038/s41467-022-28937-x)
Supplement: Supplementary file 5 — Reporting Summary [file 41467_2022_28937_MOESM5_ESM.pdf]

nature portfolio

Corresponding author(s): Pamela Cowin, Elena Spina

Last updated by author(s): Feb 9, 2022

Reporting Summary

Nature Portfolio wishes to improve the reproducibility of the work that we publish. This form provides structure for consistency and transparency in reporting. For further information on Nature Portfolio policies, see our Editorial Policies and the Editorial Policy Checklist.

Statistics

For all statistical analyses, confirm that the following items are present in the figure legend, table legend, main text, or Methods section.

n/a	Confirmed	
☐	☒	The exact sample size (*n*) for each experimental group/condition, given as a discrete number and unit of measurement
☐	☒	A statement on whether measurements were taken from distinct samples or whether the same sample was measured repeatedly
☐	☒	The statistical test(s) used AND whether they are one- or two-sided<br>*Only common tests should be described solely by name; describe more complex techniques in the Methods section.*
☒	☐	A description of all covariates tested
☐	☒	A description of any assumptions or corrections, such as tests of normality and adjustment for multiple comparisons
☐	☒	A full description of the statistical parameters including central tendency (e.g. means) or other basic estimates (e.g. regression coefficient) AND variation (e.g. standard deviation) or associated estimates of uncertainty (e.g. confidence intervals)
☐	☒	For null hypothesis testing, the test statistic (e.g. *F*, *t*, *r*) with confidence intervals, effect sizes, degrees of freedom and *P* value noted<br>*Give P values as exact values whenever suitable.*
☒	☐	For Bayesian analysis, information on the choice of priors and Markov chain Monte Carlo settings
☒	☐	For hierarchical and complex designs, identification of the appropriate level for tests and full reporting of outcomes
☒	☐	Estimates of effect sizes (e.g. Cohen's *d*, Pearson's *r*), indicating how they were calculated

Our web collection on statistics for biologists contains articles on many of the points above.

Software and code

Policy information about availability of computer code

Data collection	The confocal images were displayed with the Zen2 software

Data analysis

Flowjo v9 for flow cytometry
Fiji/ImageJ v2.0.0 for for analysis and visualization of microscopy data
Imaris v 9.5 to generate movie
Prism-GraphPad v 9.2.0 for statistics and generating graphs
Data collection for flow cytometry was done on Beckton-Dickinson (East Rutherford, NJ) LSRII analyser. Cell sorting was performed on a FACSAria IIu
RStudio v 1.2.5019 for scRNAseq analysis

RNAseq analysis used:
STAR v 2.5.0c
PICARD v 1.126
BEDTools v 2.17.0
bedGraphToBigWig tool v4
HTSeq v 0.6.0
DESeq2 v 1.10.0
iCellR v 1.5.5

GEO2R. Version info: R 3.2.3, Biobase 2.30.0, GEOquery 2.40.0, limma 3.26.8. for microarray analyses

For manuscripts utilizing custom algorithms or software that are central to the research but not yet described in published literature, software must be made available to editors and reviewers. We strongly encourage code deposition in a community repository (e.g. GitHub). See the Nature Portfolio guidelines for submitting code & software for further information.

Data

Policy information about availability of data

All manuscripts must include a data availability statement. This statement should provide the following information, where applicable:

- Accession codes, unique identifiers, or web links for publicly available datasets
- A description of any restrictions on data availability
- For clinical datasets or third party data, please ensure that the statement adheres to our policy

Data availability: The authors declare that all data supporting the findings of this study are available within the article and its supplementary information files or from the corresponding authors upon request.

All RNAseq data used in this study has been deposited in the Gene Expression Omnibus (GEO) database accession number GSE190180 and are publicly available https://www.ncbi.nlm.nih.gov/geo/query/acc.cgi?acc=GSE190180.

Previously published scRNAseq data that were re-analyzed here are available under the following accession codes
GSE109711: https://www.ncbi.nlm.nih.gov/geo/query/acc.cgi?acc=GSE109711
GSM2759554: https://www.ncbi.nlm.nih.gov/geo/query/acc.cgi?acc=GSM2759554
GSM2759555: https://www.ncbi.nlm.nih.gov/geo/query/acc.cgi?acc=GSM2759555
GSM2510617: https://www.ncbi.nlm.nih.gov/geo/query/acc.cgi?acc=GSM2510617
GSM2510616: https://www.ncbi.nlm.nih.gov/geo/query/acc.cgi?acc=GSM2510616
The mm10 reference genome: http://ftp.ensembl.org/pub/release-102/fasta/mus_musculus/dna/

Microarray data GSE3165 (https://www.ncbi.nlm.nih.gov/geo/).

The cBioPortal platform was used to access human breast cancer data derived from TCGA Pancancer and Metabric
https://www.cbioportal.org/study/summary?id=brca_tcga_pan_can_atlas_2018
https://www.cbioportal.org/study/summary?id=brca_metabric

All source data related to graphs within the figures is provided with this paper as an excel file titled “Source Data”.

Adgra3cre mice generated in this study are available via the Mutant Mouse Resource and Research Center (MMRRC) Stock No. 068344 (https://www.mmrrc.org/catalog/sds.php?mmrrc_id=68344).

Code availability: No in house codes/pipelines were used.

Field-specific reporting

Please select the one below that is the best fit for your research. If you are not sure, read the appropriate sections before making your selection.

☒ Life sciences ☐ Behavioural & social sciences ☐ Ecological, evolutionary & environmental sciences

For a reference copy of the document with all sections, see nature.com/documents/nr-reporting-summary-flat.pdf

Life sciences study design

All studies must disclose on these points even when the disclosure is negative.

Sample size	No statistical methods were used to predetermine sample size. Sample size was limited by animal availability and the ethical requirement to use the minimum numbers of animal biological replicates while ensuring reproducibility of the results
Data exclusions	No animal was excluded from this study.
Replication	All experiments have been repeated at least twice and gave identical or very similar results.
Randomization	The experiments were not randomized. Female transgenic mice of mixed background or pure FVBN background were used and compared to their respective control mice of mixed or pure FVBN background.
Blinding	At least two independent investigators have performed most of the experiments in parallel. Blinding was not possible as the same investigator processed the animals and analyzed the data. Blinding during data collection was not possible because the phenotypes resulting from genotype or hormonal manipulation produced immediately apparent histological effects on the mammary glands

Reporting for specific materials, systems and methods

We require information from authors about some types of materials, experimental systems and methods used in many studies. Here, indicate whether each material, system or method listed is relevant to your study. If you are not sure if a list item applies to your research, read the appropriate section before selecting a response.

Materials & experimental systems

n/a	Involved in the study	
☐	☒	Antibodies
☒	☐	Eukaryotic cell lines
☒	☐	Palaeontology and archaeology
☐	☒	Animals and other organisms
☒	☐	Human research participants
☒	☐	Clinical data
☒	☐	Dual use research of concern

Methods

n/a	Involved in the study	
☒	☐	ChIP-seq
☐	☒	Flow cytometry
☒	☐	MRI-based neuroimaging

Antibodies

Antibodies used	Primary antibodies:<br>Rabbit antibodies: anti-K5 (Covance, PRB160P, 1:100); anti-K14 (Abcam,Ab181595 1:100); anti-E-cadherin (Cell Signaling, 3195S, 1:100); anti-p63 (Abcam, ab124762,1:100);) anti-K14 (Covance PRB-155P 1:4000); anti-Tcf1 (Cell Signaling 22035 1:100); anti-Collagen (Rockland 1:100), anti-Fibronectin (Sigma F3648 1;100), anti-Sox11 (Millipore ABN105 1:100), anti-Ki67 (Thermo Scientific 1:100), anti-p63 (Abcam 4262 1:200); anti-progesterone receptor (PR) DAKO A0098 1:500); anti-EGFP (Life Technologies, A-11122 1:300)<br>Mouse antibodies: anti-SMA (Dako, M0851, 1:100) anti-K8 undiluted (Progen 65138); anti-E-cadherin (BD 610182 1:100); anti-PCNA (Dako M0987 1:500); anti-estrogen receptor (SRA 1010 StressGen 1:100); anti-BrdU (Invitrogen 033900 1:500) anti-p27 (Thermo Scientific Ab1 MS-256 1:200)<br>Rat antibodies: anti-K8 (Developmental Studies Hybridoma Bank, TROMA-I, 1:50)<br>Goat antibodies: anti-mP-cadherin (R&D cat # 1:100)<br><br>Secondary antibodies:<br>Alexa Fluor-conjugated antibodies from (Life technologies diluted 1:500): goat anti-mouse 647 (A21237); goat anti-rat 647 (A21247); goat anti-rabbit 647 (A21245) , donkey anti-goat 555, (A21432), Biotinlyated anti-rabbit and anti-mouse (Vector Labs, Burlingame CA diluted 1:500)<br><br>Antibodies used for flow cytometry: biotinylated- TER119 (BD 553672,1:200), biotinylated-CD31 (BD 558737,1:200), biotinylated-CD45 (BD 553077,1:200), biotinylated-CD140a (eBioscience12-1401-80,1:200), CD24-PE (BD 553262,1:400), CD49f-PerCP-Cy5.5 (Biolegend 313617,1:200,) CD49f-PE-Cy7(BD313621 1:200), CD24-FITC (BD553261,1:100), CD49f-PE (BD313611,1:100), Streptavidin-AlexaFluor647 (Molecular Probes S21374,1:600), CD61-APC (Caltag,1:200), Sca1-PE-Cy7 (eBioscience 25-5981-81,1:600), CD29-Pacific Blue (Biolegend 102224,1:200).
Validation	All antibodies are commercially available and validated for species cross-reactivity, immunostaining and/or flow cytometry specificity on their manufacturer's websites. Specificity and appropriate dilution was controlled by using sections of tissues known to express high levels of the antigen, as well as sections of tissues known to lack expression of the antigen. All immunostaining experiments

were performed along with a secondary antibody control slide, to ensure specificity.

Animals and other organisms

Policy information about studies involving animals; ARRIVE guidelines recommended for reporting animal research

Laboratory animals	All studies involving mice received ethical approval by the Institutional Animal Care and Use Committee (IACUC) at NYU School of Medicine under protocols 202000144 and TR201900137/IA16-00513. All mice were housed in individually ventilated cages under a 12:12h light–dark cycle with water and food available ad libitum and monitored daily in an ALAC accredited facility, and euthanized by CO2 anesthesia followed by cervical dislocation.<br>Males and females of the following strains were maintained as breeding stock. Female progeny only were used in all analyses:<br>Adgra3-lacZ mice from Regeneron, were backcrossed onto an FVB/N strain background and control FVB/N. Their mammary tissue was harvested during puberty (5-7 weeks of age) for flow cytometry, RNAseq, ductal elongation and hormonal deprivation/ supplementation studies. For X-Gal localization studies, tissue was harvested from nulliparous mice at day 2, day 18, weeks 3, 4, , 8, 12, 26 and 36, during early, mid and late pregnancy (P12, P13.5, P16.5, P18.5), lactation, involution days 1, 3, 5,10, 21 and from multiparous mice after 2 cycles of pregnancy and lactation (L6) and involution and from embryos at E14,E15,E18.5.<br>Adgra3cre/+ and Adgra3cre/cre mice, generated in this study, (MMRRC Stock No. 068344) were maintained on a mixed strain background and crossed to B6.Cg-Gt(ROSA)26Sortm14(CAG-tdTomato)Hze/J (JAX:007914) for lineage tracing experiments. Tracing was activated at 5 weeks as well as during pregnancy P14.5 and P19.5 and tissue was harvested from mice 48 hrs later and at developmental time points 7, 8, 24 and 36 weeks, P15, P20.5, L6. Tissue for histological analysis of Adgra3cre/+ and Adgra3cre/cre mice was harvested at 6 weeks, P12.5 and involution days 3,11 and21.<br>MMTV-Wnt1 mice (FVB.Cg-Tg(Wnt1)1Hev/J JAX Strain #002934) were crossed to the above strains. Hyperplasia were analyzed at 7-8 weeks and tumors were collected before exceeding 2cM in diameter or 5% of body weight in accordance with IACUC guidelines.<br>Lgr5-EGFP-IRES-CreER (B6.129P2-Lgr5tm1(cre/ERT2)Cle/JStrain #008875) and sSHIP-EGFP (B6.Cg-Tg(Inpp5d-EGFP)DLrr/CprJ Strain #024808) (Jax Labs) mice backcrossed onto on an FVBN background were used for immunolocalization and FACS experiments.<br>Prepubertal 3 week old homozygous CrTac:NCR-Foxn1nu(NCRNU) (Taconic) females were used as hosts for mammary cell transplantation assays. Lineage tracing was activated in Adgra3cre/ROSA-TdT donors at 5 weeks and tissue harvested 3 weeks later.
Wild animals	No wild animals were used
Field-collected samples	No field collected samples were used
Ethics oversight	All animal protocols were approved by the Institutional Animal Care and Use Committee (IACUC) at NYU School of Medicine.

Note that full information on the approval of the study protocol must also be provided in the manuscript.

Flow Cytometry

Plots

Confirm that:

- [x] The axis labels state the marker and fluorochrome used (e.g. CD4-FITC).
- [x] The axis scales are clearly visible. Include numbers along axes only for bottom left plot of group (a 'group' is an analysis of identical markers).
- [x] All plots are contour plots with outliers or pseudocolor plots.
- [x] A numerical value for number of cells or percentage (with statistics) is provided.

Methodology

Sample preparation	Mammary epithelial cell preparation<br>All reagents were purchased from Stem Cell Technologies (Vancouver, British Columbia) unless otherwise noted. Mice were sacrificed with CO2 followed by cervical dislocation, and the 3rd, 4th, and 5th mammary glands were excised, discarding the lymph nodes. Harvested glands were transferred to a sterile petri dish and finely minced between two scalpel blades into a homogeneous slurry. The homogenate was added to a 15mL tube containing a mixture of 1 mL Collagenase/Hyaluronidase and 9 mL Epicult-B Basal Medium. The tubes were incubated on a rotating platform for 6-8 hours at 37°C. The digests were then vortexed briefly and centrifuged at 450 x g for 10 minutes to pellet the epithelial organoids. Pellets was resuspended in 1mL of 0.25% Trypsin-EDTA and incubated at 37°C for 1 minute, quenched with HBSS supplemented with 2% FBS(HF), and centrifuged again at 450 x g for 6 minutes. The pellet was resuspended in 1mL Dispase with 1mg/mL DNase I (Roche, Indianapolis, IN) for 1 minute, pelleted, and resuspended in NH4Cl red blood cell (RBC) lysis buffer for 1 minute at room temperature. A single cell suspension was made by filtering through a 40µm mesh strainer (BD, East Rutherford, NJ) into 5mL HF.<br>Flow cytometry and cell sorting. To detect Gpr125- B-gal expression, cells were labelled with fluorescein di-V-galactoside (FDG) according to manufacturer's protocol (Molecular Probes, Eugene, Oregon). Mammary epithelial cells were prepared as described above. For FDG staining, cells were resuspended at 107/mL in HBSS supplemented with 2% FBS, and the samples were pre-warmed at 37°C for 10 minutes. FDG loading was performed by adding an equal volume of pre-warmed 2mM FDG (diluted in distilled water) to the cell suspension for exactly 1 minute at 37°C, then immediately quenched by adding 2mL icecold HF. The FDG-loaded cells were then centrifuged 1000 x g and stained with surface antibodies. Cells were incubated with

	conjugated antibodies diluted in HF, for 30 minutes on ice in a dark container, washed with 2mL of HF, and resuspended in 250μL HF for analysis. Cell viability was assessed by adding 4',6-Diamidino-2-phenylindole (DAPI, Sigma-Aldrich) to the final suspension at a concentration of 1μg/mL.
Instrument	Data collection for flow cytometry was done on a Beckton-Dickinson (East Rutherford, NJ) LSRII analyser. For cell sorting, cells were were sorted using FACSAria IIu
Software	Analyses were done using Flowjo v9
Cell population abundance	At least 200,000 total cells were captured in all experiments
Gating strategy	The gating strategy is described in the figure legend for each experiment.<br>Here the main gatings for all the experiments: FSC/SSC gating to discard debris, then SSC-A/SSC-W for gating singlets, DAPI/FSC to select alive cells, Lin (CD45/CD31/Ter119/CD140a)/SSC to discard hematopoietic cells, then CD24/CD49 to visualize Mammary Epithelial Cells and FDG/FSC to visualize FDG for Gpr125 positive cells.

☒ Tick this box to confirm that a figure exemplifying the gating strategy is provided in the Supplementary Information.
